# High Dimensional Immune Profiling Reveals Different Response Patterns in Active and Latent Tuberculosis Following Stimulation With Mycobacterial Glycolipids

**DOI:** 10.3389/fimmu.2021.727300

**Published:** 2021-11-23

**Authors:** Carolina S. Silva, Christopher Sundling, Elin Folkesson, Gabrielle Fröberg, Claudia Nobrega, João Canto-Gomes, Benedict J. Chambers, Tadepally Lakshmikanth, Petter Brodin, Judith Bruchfeld, Jérôme Nigou, Margarida Correia-Neves, Gunilla Källenius

**Affiliations:** ^1^ Life and Health Sciences Research Institute, School of Medicine, University of Minho, Braga, Portugal; ^2^ ICVS/3B’s, PT Government Associate Laboratory, Braga, Portugal; ^3^ Division of Infectious Diseases, Department of Medicine Solna, Center for Molecular Medicine, Karolinska Institutet, Stockholm, Sweden; ^4^ Department of Infectious Diseases, Karolinska University Hospital, Stockholm, Sweden; ^5^ Center for Infectious Medicine, Department of Medicine, Huddinge, Karolinska Institutet, Stockholm, Sweden; ^6^ Science for Life Laboratory, Department of Women’s and Children’s Health, Karolinska Institutet, Stockholm, Sweden; ^7^ Department of Immunology and Inflammation, Imperial College London, London, United Kingdom; ^8^ Institut de Pharmacologie et de Biologie Structurale, Université de Toulouse, Centre National de la Recherche Scientifique (CNRS), Université Paul Sabatier, Toulouse, France

**Keywords:** tuberculosis, mycobacterial glycolipids, active tuberculosis (ATB), latent tuberculosis (LTB), hyporesponsiveness, lipoarabinomannan (LAM), phosphatidylinositol mannoside (PIM)

## Abstract

Upon infection with *Mycobacterium tuberculosis* (Mtb) the host immune response might clear the bacteria, control its growth leading to latent tuberculosis (LTB), or fail to control its growth resulting in active TB (ATB). There is however no clear understanding of the features underlying a more or less effective response. Mtb glycolipids are abundant in the bacterial cell envelope and modulate the immune response to Mtb, but the patterns of response to glycolipids are still underexplored. To identify the CD45^+^ leukocyte activation landscape induced by Mtb glycolipids in peripheral blood of ATB and LTB, we performed a detailed assessment of the immune response of PBMCs to the Mtb glycolipids lipoarabinomannan (LAM) and its biosynthetic precursor phosphatidyl-inositol mannoside (PIM), and purified-protein derivate (PPD). At 24 h of stimulation, cell profiling and secretome analysis was done using mass cytometry and high-multiplex immunoassay. PIM induced a diverse cytokine response, mainly affecting antigen-presenting cells to produce both pro-inflammatory and anti-inflammatory cytokines, but not IFN-γ, contrasting with PPD that was a strong inducer of IFN-γ. The effect of PIM on the antigen-presenting cells was partly TLR2-dependent. Expansion of monocyte subsets in response to PIM or LAM was reduced primarily in LTB as compared to healthy controls, suggesting a hyporesponsive/tolerance pattern derived from Mtb infection.

## Introduction

It is estimated that approximately 25% of the world population is latently infected with *Mycobacterium tuberculosis* (Mtb) (1). However, only about 10% of individuals with latent TB (LTB) are estimated to develop active TB (ATB) (2). It is clear therefore that in most cases Mtb infection is well controlled, but our understanding of what makes an effective immune response that controls and/or clears Mtb is limited.

Research on the host response to Mtb has so far mainly focused on protein-based antigens. However, the immune response to Mtb is initiated mainly through the interaction of Mtb cell envelope components, mostly glycolipids, with distinct cells of the innate immune system (3), which trigger activating or repressive responses in terms of cytokine production (4, 5). The ability of Mtb lipids to traffic outside infected cells (6–8) renders the direct contact of Mtb cell envelope glycolipids with distinct immune cells an important aspect of the immune response (9). Lipoarabinomannan (LAM) is a major glycolipid of the Mtb cell wall and has been studied quite extensively for its immunomodulatory properties (10, 11), compared to its biosynthetic precursors, the phosphatidyl-inositol mannosides (PIM_2_ and PIM_6_). Many host cell receptors take part in the initial interaction between mycobacteria and innate immune cells (12, 13). TLRs and C-type lectins are involved in this process, resulting in activation of several antimicrobial mechanisms by macrophages (Mφs) and dendritic cells (DCs) (14–17).

In addition to the extensive interaction with innate immune cells, PIM and LAM are also both recognized by CD1b-restricted T cells (9, 18–21). In fact, it was observed that purified-protein derivate (PPD) positive individuals respond through CD1-restricted T cells to several mycobacterial lipids, including PIM and LAM (18) and that this response may vary between individuals with ATB and LTB. Mtb whole lipid extract was shown to induce proliferation of CD1-restricted CD4^+^ and, to a smaller extent, CD8^+^ T cells in LTB. Interestingly, the same was observed for ATB patients only after the first two weeks of anti-TB treatment (22). A subset of LAM reactive CD1-restricted T cells co-expressing perforin, granulysin, and granzyme B (GrzB), mostly CD8^+^, are more frequent in LTB than in individuals who developed ATB (evaluated after TB treatment) (22). Similarly, glycerol monomycolate-specific T cells are more frequent in LTB than ATB patients (18) and the response of these cells may vary between ATB and LTB individuals.

B cell-mediated immunity in Mtb infection has been less explored compared to monocyte- and T cell-mediated responses, although recent data strengthen the relevance of these cells in the immune response to Mtb. Recently it was shown that Mtb LAM induces IL-10 production by B cells and that these cells (B10) inhibit CD4^+^ T_H_1 polarization leading to increased Mtb susceptibility in mice (23). The response of B cells to LAM was shown to occur in a TLR2-dependent manner (23).

In the present study, we performed a detailed assessment and simultaneous comparison of the immune response to PIM, LAM and PPD from Mtb in peripheral blood mononuclear cells (PBMCs) from individuals with ATB or LTB and compared with healthy controls (HC). We performed immune profiling by secretome analysis and mass cytometry measuring simultaneously 37 cellular markers at the single-cell level to allow high-resolution of the cellular composition and secretion. We identified distinct subsets within memory T cells, NK cells, B cells and monocytes/DCs that were altered by PPD, PIM and LAM stimulation and further evaluated the role of TLR2 in this process.

## Materials and Methods

### Study Participants

Participants were recruited in 2018 within an ongoing prospective cohort of adult (≥18 years) TB patients and contacts attending the TB Centre, Dept of Infectious Diseases Karolinska University Hospital Stockholm (**Supplemental Table 1**). ATB cases were defined upon microbiological (PCR and/or culture) verification. LTB participants were defined as asymptomatic, IGRA positive, close contacts to ATB cases. Healthy controls (HC) were defined as IGRA negative students and hospital staff without known previous Mtb exposure. Exclusion criteria were pregnancy, autoimmune diseases and HIV co-infection or other immunodeficiencies. ATB and LTB participants were screened with standard biochemical set-up and radiology.

### Antigens

Tuberculin PPD (RT 50) was obtained from Statens Serum Institute, and PHA from *In vivo*gen. LAM and PIMs were prepared as previously described in detail (5). Briefly, heat-killed bacteria were frozen and thawed several times, sonicated and extracted in 40% hot phenol for 1 h at 70°C. ManLAM and PIM were obtained from the water and phenol phases respectively. The dialyzed water phase was submitted to affinity chromatography on Concanavalin A-Sepharose. After elution, bound material was subjected to hydrophobic interaction chromatography on Phenyl-Sepharose (Amersham, Sweden). Bound ManLAM was eluted and further separated from other glycolipids by gel filtration on Sephacryl S-100 (Amersham, Sweden). The phenol phase obtained above was washed 3 times with PBS and extracted with an equal volume of 2% SDS in PBS overnight at room temperature. The resulting water phase was precipitated with of ice-cold ethanol. PIMs contained in the precipitate were purified to homogeneity by gel filtration on Sephacryl S-100. PIM contains both PIM_2_ and PIM_6_ isoforms, differing in number of fatty acyl constituents (5).

### PBMC Isolation

Venous blood from each participant was collected into EDTA tubes and PBMCs were purified through density gradient centrifugation using Lymphoprep™ (Stemcell) according to the manufacturer’s instruction, with some modifications. The cell isolation primarily removes granulocytes and red blood cells. Briefly, white blood cells were counted using a HemoCue instrument and the blood was diluted to a maximum of 240x10^6^ cells per 22.5 ml that were then layered onto 10 ml Lymphoprep. The cells were centrifuged at 400*g* for 30 min without any break. The mononuclear cell layer was collected into a new 50 ml tube and resuspended to 45 ml with PBS. The cells were spun at 300g for 10 min with break after which the cells were resuspended into 1-5 ml PBS and filtered using a 100 μm poor size cell strainer and counted on a Countess (ThermoFisher Scientific). The cells were centrifuged at 400*g* for 10 min with break and resuspended with freeze media (90% FBS supplemented with 10% DMSO) and placed in a CoolCell freezing container (Sigma) before moving to –80°C overnight followed by long-term storage in liquid nitrogen.

### PBMC Stimulation

PBMCs from 5 patients with ATB, 5 with LTB and five HCs were thawed at 37°C followed by addition of 1 mL RPMI-1640 media supplemented with 10% fetal bovine serum (FBS), 1% penicillin/streptomycin (P/S) and 250 U/mL Benzonase (all from ThermoFisher). The cells were washed twice (300*g* for 5 min) in media followed by resuspension in RPMI-1640 culture media supplemented with 10% FBS, 1% P/S, 0.3 g/L L-Glutamine and 25 mM HEPES and counted. The cells were then plated in 24-well plates at 2x10^6^ PBMCs/mL in culture media containing either 5 μg/mL PHA, 10 μg/mL PPD, or 25 μg/mL LAM or PIM, or left untreated (PBS), for 24 h in a 37°C 5% CO_2_ incubator. 4 h before collection, 5 μg/mL of brefeldin A and 2 μM Monensin (both ThermoFisher) were added to each well. PHA was used as positive control for PBMCs responsiveness (**Supplemental Figures 1**, **2**). The choice of concentration of LAM and PIM was based on titrations with cytokine secretion into supernatants as read-out (data not shown). The 24 h stimulation did not alter cell numbers between the conditions (**Supplemental Figure 3**).

### Mass Cytometry Staining and Acquisition

After 24 h, cells were collected by centrifugation after a 15 min incubation with 2 mM EDTA. Supernatants were stored at –80°C and cells were fixed using the PBMCs fix kit (Cytodelics AB) and barcoded using Cell-ID™ 20-Plex Pd Barcoding Kit (Fluidigm Inc.), according to the manufacturer’s recommendations. Samples were washed with CyFACS buffer (PBS with 0.1% BSA, 0.05% sodium azide and 2mM EDTA) and Fc receptors were blocked with 200 μL of blocking buffer (Cytodelics AB) for 10 min at RT. Cells were incubated with 200 μL of antibody cocktail (**Supplemental Table 2**) for 30 min at 4°C, washed with CyFACS buffer, and fixed with 1% formaldehyde. For intracellular staining, cells were permeabilized using an intracellular fixation and permeabilization kit (eBiosciences Inc.) according to the manufacturer’s instructions. Subsequently, 200 μl of intracellular antibody cocktail (**Supplemental Table 3**) was added and incubated for 45 min at RT. Cells were washed, fixed in 4% formaldehyde at 4°C overnight, and stained with DNA intercalator (0.125 µM MaxPar^®^ Intercalator-Ir, Fluidigm Inc.) on the following day. After that, cells were washed with CyFACS buffer, PBS and MiliQ water, counted and adjusted to 750,000 cells/mL. Samples were acquired in a CyTOF2 (Fluidigm) mass cytometer at a rate of 250-400 events/s using CyTOF software version 6.0.626 with noise reduction, a lower convolution threshold of 200, event length limits of 10-150 pushes, a sigma value of 3, and flow rate of 0.045 ml/min.

### Analysis of Mass Cytometry Data

The mass cytometry FCS data files were gated for different cell subsets: CD45^+^ leucocytes, CD45^+^CD3^+^CD20^–^ T cells, CD45^+^CD3^–^CD7^+^ NK cells, CD45^+^CD3^–^HLA-DR^+^ antigen-presenting cells (APCs), and CD45^+^ leukocytes producing IL-2, IL-4, IL-5, IL-6, IL-10, IL-17A, IFN-γ, TNF-α, GrzB, and GM-CSF using FlowJo™ v10.6.1. The gated populations were exported to new FCS files that were then analyzed using the R-package Cytofkit v1.12.0, which includes an integrated pipeline for mass cytometry analysis (24). Cytofkit was run in R-studio version 1.1.463 and R version 3.6.1. For analysis of total leukocytes, 5000 cells were used per sample. For analysis of gated T cells, NK cells, and APCs, 10000 cells were used per sample. For analysis of cytokine^+^ cells, a ceiling of 5000 cells were included per sample. Dimensionality was reduced using Barnes-Hut tSNE with a perplexity of 30 with a maximum of 1000 iterations. Clustering was then performed using density-based machine learning with ClusterX (24) and cell subsets were identified by visual inspection of marker expression for each cluster. The Cytoftkit analysis was performed using PBS, PPD, PIM, and LAM FCS files together, whereas PHA stimulated cells were evaluated independently, using only PBS and PHA FCS files.

### Secretome Analysis of Culture Supernatants

Cell culture supernatants (n=75) were randomized in a 96-well plate and analyzed with a multiplex proximity extension assay (PEA) (25), enabling simultaneous quantification of 92 inflammatory markers from the Olink inflammation panel (**Supplemental Table 4**). Markers where all samples were below the limit of detection of the assay were removed from subsequent analysis. The samples were run by the Translational Plasma Profile Facility at SciLifeLab, Stockholm, Sweden.

### TLR2-Dependence of PBMCs Activation

To investigate TLR2-dependent PBMCs activation by the Mtb glycolipids PIM and LAM, frozen PBMCs from HC (n=5) were thawed in a 37°C water bath, washed 2 times in complete media (RPMI-1640 culture media supplemented with 10% FBS, 1% P/S, 1 mM sodium pyruvate and 10 mM HEPES) and plated as described for mass cytometry. Prior to stimulation, the cells were pre-incubated for 30 min at 37°C with 5 μg/mL of anti-TLR2 monoclonal antibody (clone T2.5, *In vivo*Gen) or with an isotype control (mIgG1, eBiosciences).

### Flow Cytometry

Cells stimulated in the presence or absence of anti-TLR2 antibody for 24 h were collected after an additional 15 min incubation with 2 mM EDTA. The cells were then washed with FACS buffer (PBS with 0.3% BSA and 2 mM EDTA) and Fc receptors were blocked with 20 μL of blocking buffer Fc Receptor Binding Inhibitor (eBiosciences) for 10 min at 4°C. The cells were incubated with 50 μL of antibody cocktail (**Supplemental Table 5**) for 30 min at RT, washed with PBS and incubated with Fixable Viability Dye eFluor™ 450 (eBiosciences) for 30 min at 4°C. For intracellular staining, the cells were permeabilized using the FoxP3 intracellular fixation and permeabilization kit (eBiosciences) according to the manufacturer’s instructions. Subsequently, 50 μl of intracellular Ab cocktail (**Supplemental Table 5**) was added and incubated for 30 min at 4°C. Finally, the cells were washed, resuspended in PBS and kept at 4°C until acquisition on the next day. The cells were acquired on a 12-color LSRII flow cytometer using FACSDiva software (Becton Dickinson, Franklin Lakes, NJ); data analysis was performed using FlowJo™ v10.6.1. Gating strategies are represented in **Supplemental Figure 4**.

### Statistical Analysis

Comparisons of a single variable for paired data for >2 groups were evaluated by Friedman’s test followed by Dunnet’s *post-hoc* test. Comparisons of a single variable for unpaired data for >2 groups were evaluated by using a Kruskal-Wallis test followed by Dunn’s post-test. Comparisons of >1 variable for paired data were evaluated using repeated measures 2-way ANOVA followed by Dunnett’s *post-hoc* test. Differences were considered significant when p<0.05. Statistical analyzes were performed using Prism9 (GraphPad Software, USA).

### Study Approval

Written informed consent was received from all participants before inclusion in the study, whereby they were pseudoanonymized. The study was approved by the Regional Ethical Review Board at the Karolinska Institute in Stockholm (approval numbers 2013/1347-31/2 and 2013/2243-31/4) and by the Ethics Committee for Research in Life and Health Sciences of the University of Minho, Portugal (approval number SECVS 014/2015) and it is in accordance with the Declaration of Helsinki.

## Results

### Effect of Stimulants on Cell Types and Cytokine Production

**Figure 1:**
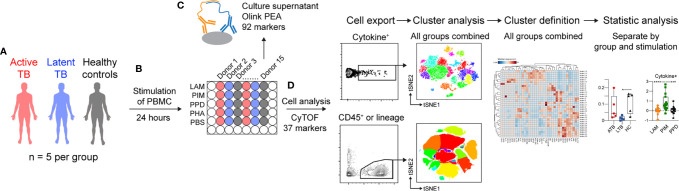
Schematic of the study outline. **(A)** PBMCs from three groups, including active TB (ATB, red), latent TB (LTB, blue) and healthy controls (HC, black) were **(B)** stimulated for 24 h with the the mycobacterial glycolipids LAM or PIM. Control stimulations included PPD, or phytohemagglutinin (PHA), or mock stimulation (PBS). **(C)** Culture supernatants were analyzed by proximity extension assay (PEA), while **(D)** cells were analyzed by mass cytometry. Cells were either pre-gated for cytokine-secreting cells or different cell subsets prior to dimensionality reduction and cluster analysis. *p<0.05, ***p<0.001, ****p<0.0001.

To investigate the effect of the Mtb glycolipids LAM and PIM on the immune response, PBMCs from individuals with ATB or LTB, and HC (**Supplemental Table 1**) were thawed and stimulated for 24 h; PPD was used as a control for responses to Mtb proteins, while mock stimulation (PBS) or phytohemagglutinin (PHA) were used as negative and positive culture controls, respectively. Proteins released into the culture supernatant were analyzed using the Olink proximity-extension assay (PEA), that allows for simultaneous measurement of 92 inflammatory markers. Cells were analyzed using mass cytometry for changes in the expression of 27 surface and 10 intracellular markers (**Figure 1** and **Supplemental Tables 2**, **3**).

**Figure 2:**
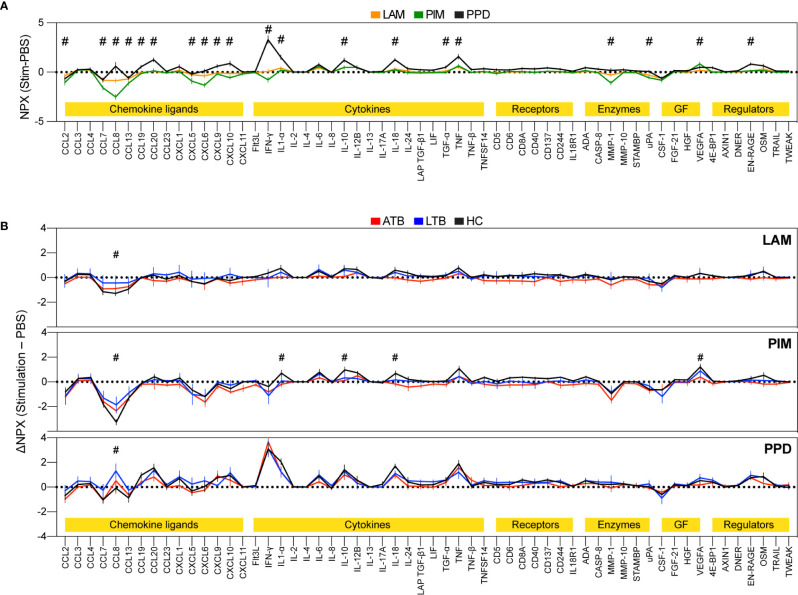
Secretion of inflammatory proteins after stimulation with LAM, PIM, and PPD. Culture supernatants were assessed for 92 protein markers using the Olink inflammation panel after 24 h of culture. Only markers above background levels were included (n=58). The graphs show normalized protein expression (ΔNPX) for stimulated (LAM, PIM, or PPD) minus unstimulated (PBS) PBMCs (**A**, n=15/group) and separated into individuals with active TB (ATB; red), latent TB (LTB, blue), and healthy controls (HC, black) (**B**, n=5/group). The lines indicate the mean and error bars indicate SEM. The protein markers were grouped into different functional groups (yellow highlight), where GF correspond to growth factors. Statistics was evaluated using two-way ANOVA followed by Dunnet’s posthoc test with significant differences between ATB or LTB against HC indicated by #, corresponding to a p-value < 0.05.

To assess the effect of LAM, PIM, and PPD on secretion of cytokines and chemokines from stimulated PBMCs, we assessed the culture supernatant for relative levels of 92 different soluble inflammatory markers (**Figure 2**). Of these, we observed changes in protein levels for 58 proteins. LAM and PIM stimulation produced very similar marker profiles, with a slightly stronger effect from PIM, suggesting a similar mechanism of action. PPD induced a markedly different response, with considerably higher IFN-γ levels, but also several other inflammatory proteins, such as IL1α and CCL8, compared to LAM and PIM, suggesting a different mechanism of action (**Figure 2A**).

There were also some indications of different levels of secretion between the groups (ATB/LTB/HC), primarily with a greater effect observed for HC compared with ATB or LTB (**Figure 2B**). IL-1α, IL-10, IL-18, and VEGF were detected at higher levels in HC compared with LTB or ATB while CCL8 was significantly decreased in HC compared with ATB and/or LTB. CCL8 functions as a strong monocyte chemoattractant but has also been associated with multiple other effects on leukocyte behavior, suggesting that its lower levels could be due to its increased uptake from the culture supernatant by activated monocytes (26). Another possibility is that it is produced to a larger extent in PPD stimulated cultures, potentially *via* synergistic effects from IFN-γ and IL-1 as has been proposed previously (27).

### Intracellular Cytokine Production in Response to Stimulation

**Figure 3:**
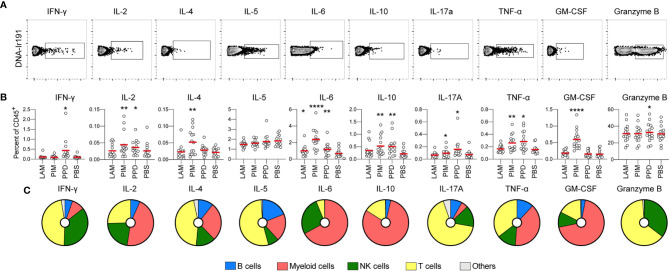
Stimulation-induced cytokine production. **(A)** Representative gates for identification of cytokine producing CD45^+^ cells *via* intracellular staining. **(B)** The frequency of cytokine positive cells out of CD45^+^ cells for each stimulation at 24h. All donors merged (n=15/stimulation). Statistics was evaluated by Friedman’s test with Dunnet’s posttest where every group was compared with unstimulated cells (PBS). *p<0.05, **p<0.01, ****p<0.0001. **(C)** For each cytokine, the pie-charts indicate the frequency of cell type responsible for its production: B cells (blue), myeloid cells (red), NK cells (green), T cells (yellow), and others (grey).

To evaluate the effect of each stimulus on intracellular production of cytokines and GrzB, regardless of the experimental group, the cumulative frequency of cytokine^+^ cells among CD45^+^ leukocytes was compared to that of unstimulated cells (PBS; **Figures 3A, B**). As expected, PPD stimulation resulted in an increase in IFN-γ-producing cells, but also led to higher levels of IL-2, IL-6, IL-10, IL-17A, TNF-α and GrzB-producing cells. Unlike PPD, PIM did not stimulate production of IFN-γ but instead stimulated early production of IL-4 and GM-CSF. In addition, PIM stimulation led to increased levels of IL-2^+^, IL-6^+^, IL-10^+^, IL17A^+^ and TNF-α^+^ cells (**Figure 3B**). To better understand if the cytokines were produced one their own or in combinations, we assessed polyfunctionality of the stimulated cells using the R-package COMPASS (28). Since GrzB is functionally distinct from the cytokines, it was excluded from the analysis. In total, 512 different combinations of cytokines, as defined by a Boolean gating strategy in FlowJo were included in the analysis. We observed that all stimulations resulted in polyfunctional cytokine production, although responses to LAM were significantly lower than to PIM and PPD (**Supplemental Figure 5**).

To get an overview of which cell types that were responsible for the cytokine production, we identified the cell subsets producing each cytokine, regardless of the group and stimuli. We observed that myeloid cells (identified through the expression of CD33) contributed strongly to the early production of IL-2, IL-6, IL-10, TNF-α, and GM-CSF. This is consistent with myeloid cells being the main source of pro-inflammatory cytokines such as IL-6 and TNF-α (29) (**Figure 3C**). This pattern largely overlapped with the cytokines stimulated by PIM, indicating that myeloid cells could be the main effector cells stimulated by Mtb glycolipids. T cells were the main cytokine producers of IFN-γ, IL-4, IL-5, IL-17A, and GrzB, while NK cells primarily produced IFN-γ, IL-2, IL-6, IL-17A, TNF-α, and GrzB. We also identified B cells, producing primarily IL-2, IL-4, IL-5, IL-17A, and TNF-α, although to a smaller extent compared with the other cell subsets (**Figure 3C**).

In summary, stimulation with Mtb glycolipids and PPD led to a polyfunctional cytokine response associated with production from multiple cell subsets.

### Reduced Cytokine Production in Individuals With Active- or Latent TB

**Figure 4:**
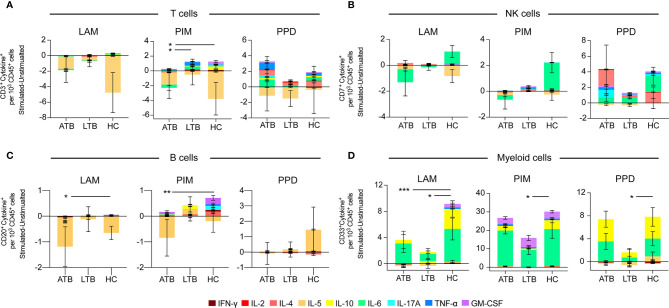
Overall effect of stimulation on cytokine production from individuals with active- and latent TB and healthy controls. Average number of cytokine^+^ cells following 24 h of stimulation with LAM, PIM, or PPD of PBMCs from individuals with active TB (ATB), latent TB (LTB), or healthy controls (HC) among **(A)** CD3^+^ T cells, **(B)** CD3^–^CD7^+^ NK cells, **(C)** CD20^+^ B cells, and **(D)** CD33^+^ myeloid cells. The groups average number of cytokine-producing cells were compared using the Friedman test followed by Dunn’s posttest. *p<0.05, **p<0.01, ***p<0.001.

To investigate the overall cytokine response profile of the main cell populations in individuals with ATB, LTB and HC, we pooled all the cytokine-producing cells of each cell population after subtracting the number in unstimulated conditions for each donor and compared the cumulative production of cytokines within the different groups (**Figure 4**). For T cells, we observed a reduced cytokine production in individuals with ATB and LTB to PIM stimulation (**Figure 4A**). There was no overall significant effect on cytokine^+^ NK cells associated with Mtb-infection (**Figure 4B**). For B cells, the overall cytokine production was reduced in individuals with ATB upon LAM and PIM stimulation compared with HC, primarily due to a reduced production of IL-5 (**Figure 4C**). For myeloid cells, a similar reduction of cytokine^+^ cells was observed in individuals with LTB to LAM, PIM, and PPD stimulations. This effect was mainly attributed to a reduced production of IL-10 and IL-6. For ATB this effect was only observed in response to LAM (**Figure 4D**).

In summary, individuals with ATB or LTB responded with less cytokine production by especially myeloid cells and somewhat by B and T cells upon stimulation with Mtb antigens.

To further analyze the effect of LAM, PIM, and PPD on cytokine production by individual cell subsets between the three groups (ATB, LTB, and HC), we proceeded with dimensionality reduction using t-stochastic neighbour embedding (t-SNE) and cluster analysis. This was performed by pooling all PBS, PPD, PIM, and LAM mass cytometry data files together followed by analysis using Cytofkit (24). To allow a high level of resolution in the analysis, cytokine producing CD45^+^ cells were gated for the individual cytokines (see gates in **Figure 3A**) which were then analyzed separately (**Supplemental Figure 6**, **7**).

### Qualitatively Different T Cell Responses to PIM and PPD

**Figure 5:**
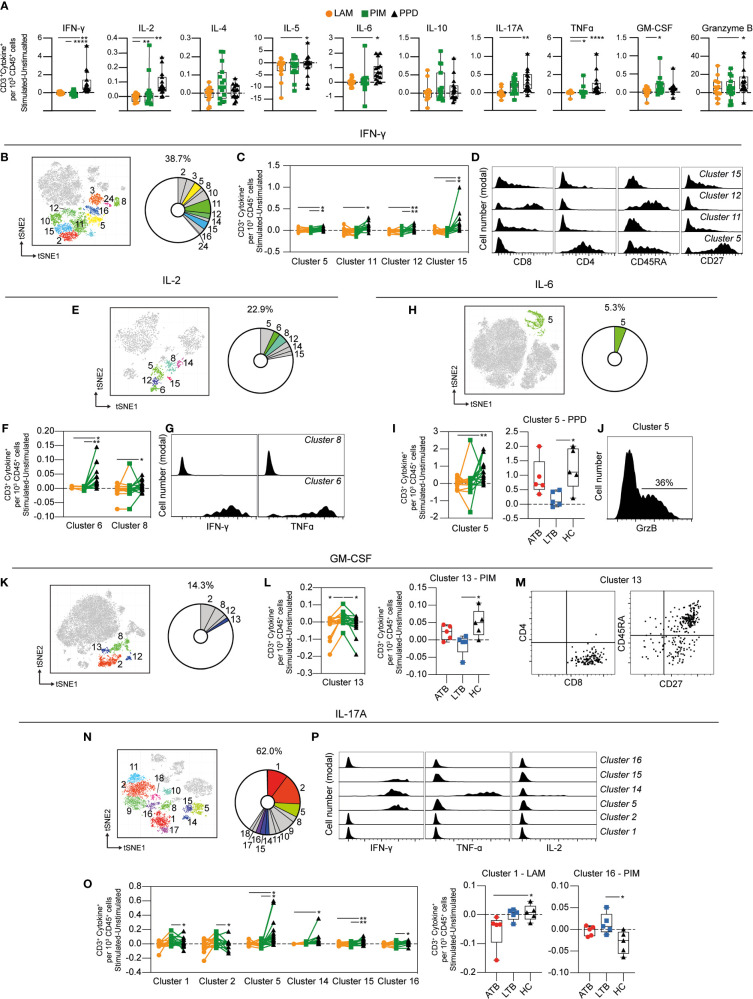
Cytokine production by stimulated T cells. **(A)** The number of cytokine-producing T cells per 1000 total CD45^+^ cells for each stimulation at 24 h with the background (unstimulated) cytokine-production removed. **(B)** Cluster analysis of IFN-γ secreting cells with clusters 2, 3, 5, 8, 10, 11, 12, 14, 15, 16, and 24 corresponding to T cells. **(C)** Clusters significantly affected by stimulation with **(D)** cluster histograms indicating CD4, CD8, CD45RA, and CD27. **(E)** Cluster analysis of IL-2 secreting cells with clusters 5, 6, 8, 12, 14, and 15 corresponding to T cells. **(F)** Clusters significantly affected by stimulation with **(G)** cluster histograms indicating IFN-γ and TNF-ɑ secretion. **(H)** Cluster analysis of IL-6 secreting cells with cluster 5 corresponding to T cells. **(I)** Cluster 5 is significantly affected by stimulation (left) and comparison of PPD stimulation on donors with active TB (ATB), latent TB (LTB) and healthy controls (HC) in cluster 5 (right). **(J)** Cluster histogram indicating GrzB secretion. **(K)** Cluster analysis of GM-CSF secreting cells with cluster 2, 8, 12, and 13 corresponding to T cells. **(L)** Cluster 13 is significantly affected by stimulation (left) and comparison of PIM stimulation on donors with ATB, LTB and HC in cluster 13 (right). **(M)** Cluster dot plots indicating CD4, CD8, CD45RA, and CD27 expression. **(N)** Cluster analysis of IL-17A secreting cells with cluster 1, 2, 5, 8, 9, 10, 11, 14, 15, 16, 17, and 18 corresponding to T cells. **(O)** Clusters significantly affected by stimulation (left) and comparison of PIM and LAM stimulation on donors with active ATB, LTB and HC in clusters 1 and 16, respectively (right) **(P)** Cluster histograms indicating IFN-γ, TNF-ɑ, and IL-2. Statistical differences between stimulations in **(A, I, L)** were evaluated by Friedman’s test with Dunnet’s posttest, while comparisons within multiple clusters (**C, F, O** left panels) were evaluated by a matched-pair two-way ANOVA with Geissner-Greenhouse correction followed by Tukey’s posttest (n=15/stimulation). Groups (ATB/LTB/HC) (**I, L, O** right panels) were compared using Kruskal-Wallis with Dunn’s posttest (n=5/group) *p<0.05, **p<0.01, ***p<0.001, ****p<0.0001.

Stimulation with PPD resulted in an increased number of T cells (identified as CD3^+^) producing IFN-γ, IL-2, IL-5, IL-6, IL-17A, TNF-α, and GrzB compared with LAM and/or PIM. Stimulation with PIM contributed to higher numbers of IL-2^+^, TNF-α^+^, and GM-CSF^+^ T cells compared with LAM stimulated cells (**Figure 5A**). Of all cells producing IFN-γ at 24 h, T cells represented 39%, comprising 11 different clusters (clusters 2, 3, 5, 8, 10, 11, 12, 14 15, 16, and 24) (**Figure 5B**). Four of these clusters (clusters 5, 11, 12, and 15) were significantly elevated by PPD stimulation compared to PIM and/or LAM (**Figure 5C**). These clusters corresponded to different CD4^+^ and CD8^+^ T cells subsets, including central memory (CD4^+^CD45RA^–^CD27^+^, cluster 5), effector memory (CD45RA^–^CD27^–^, clusters 11 and 15), and effector memory T cells re-expressing CD45RA (TEMRA - CD8^+^CD45RA^+^CD27^–^, cluster 12; **Figure 5D**).

Approximately 23% of all IL-2 producing cells were identified as T cells (**Figure 5E**). These cells comprise six clusters, of which two (clusters 6 and 8) were significantly higher following PPD stimulation compared with LAM and/or PIM (**Figure 5F**). Cluster 6 corresponded to polyfunctional CD4^+^ T cells, co-producing IFN-γ and TNF-α, while cluster 8 was composed of cells producing only IL-2 (**Figure 5G**).

Although the regulatory effect of IL-6 on T cells is well known (30), the literature on IL-6 producing T cells is limited. We identified one cluster of IL-6^+^ T cells (cluster 5) corresponding to 5.3% of total IL-6^+^ leukocytes after 24 h of stimulation (**Figure 5H**). This cluster was significantly elevated by PPD stimulation compared to LAM and was mainly attributed to ATB and HC, but not LTB individuals (**Figure 5I**). Cluster 5 was a mixed cluster consisting of cells expressing CD8^+^, CD4^+^, and double negative (DN) T cells (data not shown) with 36% of the cells co-producing GrzB (**Figure 5J**).

Approximately 14% of the GM-CSF^+^ cells were T cells, represented by four different clusters (**Figure 5K**). Cluster 13 was significantly increased upon PIM stimulation compared to LAM and PPD (**Figure 5L**). The effect was more prominent in HC individuals compared to LTB. This cluster corresponded mostly to naïve (CD45RA^+^CD27^+^) CD8^+^ T cells (**Figure 5M**).

T cells represented 62% of the IL-17A^+^ cells (**Figure 5N**). Four out of 12 clusters (clusters 5, 14, 15, and 16) were increased by PPD compared with PIM and/or LAM stimulations, while clusters 1 and 2 were increased by PIM compared with PPD (**Figure 5O** left). In addition, the analysis of individual clusters showed that stimulation with LAM reduced cluster 1 in ATB, compared with HC individuals. Also, cluster 16 was higher in LTB compared with HC upon PIM stimulation (**Figure 5O** right). Three of these clusters corresponded to polyfunctional T cells (clusters 5, 14, and 15), with clusters 5 and 15 co-producing IFN-γ, and cluster 14 co-producing IFN-γ and TNF-α (**Figure 5P**).

In summary, T cell responses were mainly observed upon stimulation with PPD. The T cells producing IFN-γ, IL-2, IL-6, and IL-17A, some of those with a polyfunctional phenotype, were significantly increased with PPD compared with LAM and/or PIM. Interestingly, however, PIM stimulation led to an increase in GM-CSF-producing T cells, particularly in HC individuals, potentially indicating a different mechanism for GM-CSF induction also associated with disease status.

### NK Cells Are Primarily Stimulated by PPD

**Figure 6:**
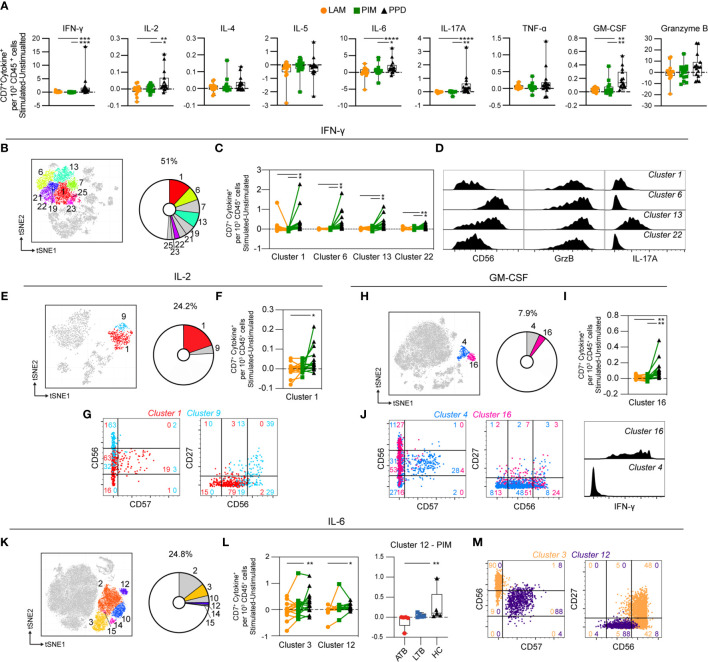
Cytokine production by stimulated NK cells. **(A)** The number of cytokine-producing NK cells per 1000 total CD45^+^ cells for each stimulation at 24 h with the background (unstimulated) cytokine-production removed. **(B)** Cluster analysis of IFN-γ secreting cells with clusters 1, 6, 7, 13, 19, 21, 22, 23, and 25 corresponding to NK cells. **(C)** Clusters significantly affected by stimulation with **(D)** cluster histograms indicating CD56 expression and GrzB and IL-17A secretion. **(E)** Cluster analysis of IL-2 secreting cells with cluster 1 and 9 corresponding to NK cells. **(F)** Clusters significantly affected by stimulation with **(G)** cluster dot plots showing CD56, CD57, and CD27 expression and histograms indicating IL-6 production. **(H)** Cluster analysis of GM-CSF secreting cells with cluster 4 and 16 corresponding to NK cells. **(I)** Cluster 16 significantly affected by stimulation. **(J)** Cluster’s dot plots showing CD56, CD57, and CD27 expression and histogram indicating IFN-γ secretion. **(K)** Cluster analysis of IL-6 secreting cells with cluster 1 and 9 corresponding to NK cells. **(L)** Clusters significantly affected by stimulation (left) and comparison of PIM stimulation on donors with active TB (ATB), latent TB (LTB) and healthy controls (HC) in cluster 12 (right). **(M)** Cluster’s dot plots showing CD56, CD57, and CD27 expression. Numbers in dot plots indicate the percentage within the cluster. Statistical differences between stimulations in **(A, F, I)** were evaluated by Friedman’s test with Dunnet’s posttest, comparisons within multiple clusters **(C, L** left**)** were evaluated by a matched-pair two-way ANOVA with Geissner-Greenhouse correction followed by Tukey’s posttest (n=15/stimulation). Groups (ATB/LTB/HC) **(L,** right**)** were compared using Kruskal-Wallis with Dunn’s posttest (n=5/group) *p<0.05, **p<0.01, ***p<0.001, ****p<0.0001.

As for T cells, the NK cells (identified as CD3^–^CD7^+^) showed minor responses to PIM and LAM, and were mainly affected by PPD stimulation, which resulted in significantly higher numbers of NK cells producing IFN-γ, IL-2, IL-6, IL-17A, and GM-CSF, compared to PIM and LAM (**Figure 6A**). Most of IFN-γ producing cells at 24 h of stimulation were NK cells. They represented 51% of all IFN-γ-producing cells and could be further divided into 9 clusters (clusters 1, 6, 7, 13, 19, 21, 22, 23, and 25) (**Figure 6B**). Of these, four clusters were significantly increased following PPD stimulation, compared with LAM and PIM (**Figure 6C**). All of these clusters were CD57^–^ but expressed different levels of CD56 suggesting that they belonged to different NK subsets. Moreover, all clusters expressed GrzB while cluster 13 also expressed IL-17A (**Figure 6D**).

IL-2-producing NK cells constituted 24% of all IL-2^+^ cells and represented two clusters (cluster 1 and 9) (**Figure 6E**). Cluster 1 was significantly increased by PPD, compared with LAM stimulation (**Figure 6F**). Both clusters were CD57^–^ while cluster 1 expressed intermediate levels of CD56 and no CD27 while cluster 9 expressed high levels of CD56 and CD27 (**Figure 6G**). Both clusters co-produced IL-6 (**Figure 6G**). NK cells represent approximately 8% of all GM-CSF-producing cells at 24 h of stimulation (**Figure 6H**). The cytokine was produced by two clusters (4 and 16), one of which (cluster 16) was significantly higher in response to PPD compared with LAM and PIM stimulation (**Figure 6I**). Similar to PPD-mediated IL-2 secreting NK cells, GM-CSF was primarily produced by CD57^–^ NK cells where >50% expressed intermediate CD56 levels while almost no cells expressed CD27 (**Figure 6J**). Cluster 16 cells were also co-producing IFN-γ (**Figure 6J**). Approximately 25% of all IL-6-producing cells at 24 h were identified as NK cells (**Figure 6K**), and two out of the six clusters (3 and 12) were significantly increased in numbers by PPD stimulation compared to LAM (**Figure 6L**). These two clusters belonged to different NK subsets with cluster 3 corresponding to CD56^high^CD57^–^ NK cells, of which 48% also expressed CD27, while cluster 12 was composed of CD56^int^CD57^+^CD27^–^ NK cells (**Figure 6M**). The IL-17A producing NK cells were composed of two clusters at 24 h. However, they were not significantly different between the different stimulations (data not shown).

Thus, similar to T cells, NK cells were primarily induced to secrete cytokines through stimulation with PPD compared with the Mtb glycolipids LAM and PIM. The stimulation led to cytokine production by CD56^int^ and CD56^bright^ NK cells, independent on the expression of CD57. In summary, these results show that stimulation with PPD leads to rapid activation of different NK cell subsets with production of primarily pro-inflammatory cytokines.

### Atypical B Cells Are a Major Source of Polyfunctional Cytokine Responses Following PIM Stimulation

**Figure 7:**
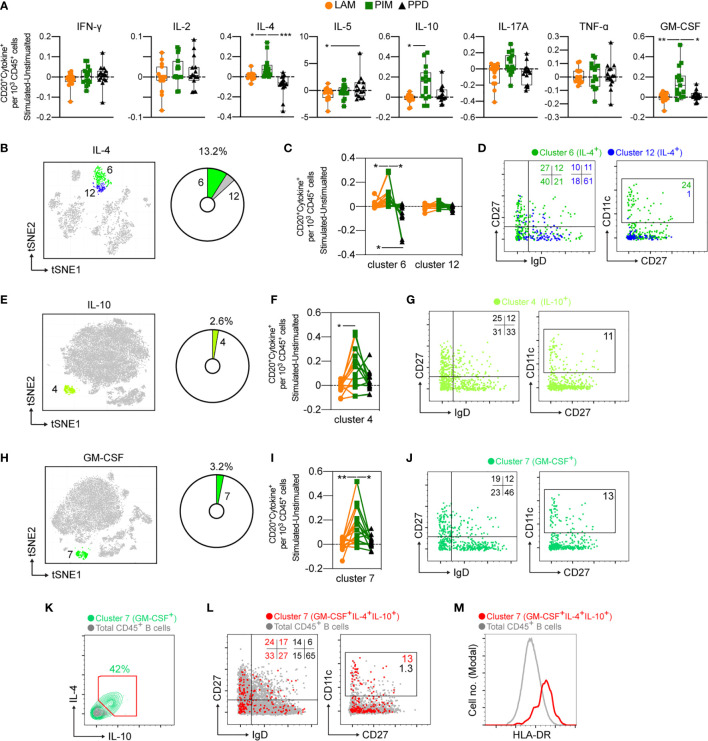
Cytokine production by stimulated B cells. **(A)** The number of cytokine positive B cells per 1000 total CD45^+^ cells for each stimulation at 24 h with the background (unstimulated) cytokine-production removed. **(B)** Cluster analysis of IL-4 secreting cells with cluster 6 and 12 corresponding to B cells. The pie-chart indicates cluster-specific and percent of total contribution to all IL-4 secreting cells. **(C)** Evaluation of the effect of LAM (orange circles), PIM (green boxes), or PPD (black triangles) stimulation on IL-4 secreting B cell clusters. **(D)** Overlay of concatenated IL-4 secreting B cells for cluster 6 (green) and cluster 12 (blue) assessing IgD and CD27 or CD11c and CD27 surface expression. **(E, G)** A similar analysis for IL-10 secreting cells and **(H–J)** GM-CSF secreting cells. **(K)** IL-4 and IL-10 co-expression among GM-CSF^+^ B cells (green) and total B cells (grey). **(L)** Overlay scatter plot of GM-CSF^+^IL-4^+^IL-10^+^ triple-secreting B cells (red) and total B cells (grey) assessing IgD and CD27 or CD11c and CD27 surface expression, with frequency of cells included in the gates indicated. **(M)** Overlay histogram indicating HLA-DR expression for GM-CSF^+^IL-4^+^IL-10^+^ triple-secreting B cells (red) and total B cells (grey). Numbers in dot plots indicate the percentage within the cluster. Statistical differences between stimulations in individual groups **(A, F, I)** were evaluated by Friedman’s test with Dunnet’s posttest, while comparisons within multiple clusters **(C)** were evaluated by a matched-pair two-way ANOVA with Geissner-Greenhouse correction followed by Tukey’s posttest (n=15/stimulation). *p<0.05, **p<0.01, ***p<0.001. n=15 for each group. Scatter and overlay plots show data concatenated from all samples and donors (n=60).

Compared with T cells and myeloid cells, B cells (defined as CD3^–^HLA-DR^+^CD20^+^) were minor producers of the measured cytokines (**Figure 3**). There was however a primarily PIM-derived effect leading to significantly increased numbers of IL-4, IL-10, and GM-CSF producing B cells in comparison to LAM and/or PPD stimulation (**Figure 7A**). There were two B cell clusters producing IL-4 (cluster 6 and 12) (**Figure 7B**), but only cluster 6 was significantly increased by PIM stimulation, with PPD leading to the lowest numbers of cells in this cluster (**Figure 7C**). Cluster 6 was enriched for switched memory (CD27^+^IgD^–^) and double negative (DN; CD27^–^IgD^–^) B cells, while cluster 12 was enriched for naïve B cells (CD27^–^IgD^+^) (**Figure 7D**). Cluster 6 was further enriched for CD11c^+^ B cells, which are associated with recent B cell activation and formation of atypical B cells during infection or inflammatory conditions (31).

B cells producing IL-10 and GM-CSF were also significantly expanded by PIM stimulation (**Figures 7E–J**). As B cells responding to PIM stimulation presented a highly homogenous phenotype, we further evaluated the cells for co-expression of the three cytokines (**Figure 7K**). We found that 42% of GM-CSF-producing B cells also produced IL-4 and IL-10. Compared with total B cell populations, the phenotype of the polyfunctional cells was highly enriched for double negative (DN - IgD^–^CD27^–^) B cells but also for unswitched and switched memory B cells (CD27^+^) compared with total B cell populations (**Figure 7L**). The polyfunctional B cells were also approximately 10-fold enriched for CD11c^+^ B cells compared with total B cells, suggesting that atypical B cells can respond to PIM stimulation (**Figure 7L**). We also quantified the levels of HLA-DR on the cell surface of the polyfunctional B cells and compared with the levels on total B cells and found an increased expression of HLA-DR on cells from cluster 7 (**Figure 7M**), consistent with previous reports on atypical B cells in mice and humans (32, 33).

### Rapid Polyfunctional Response of Myeloid Cells to PIM Stimulation

**Figure 8:**
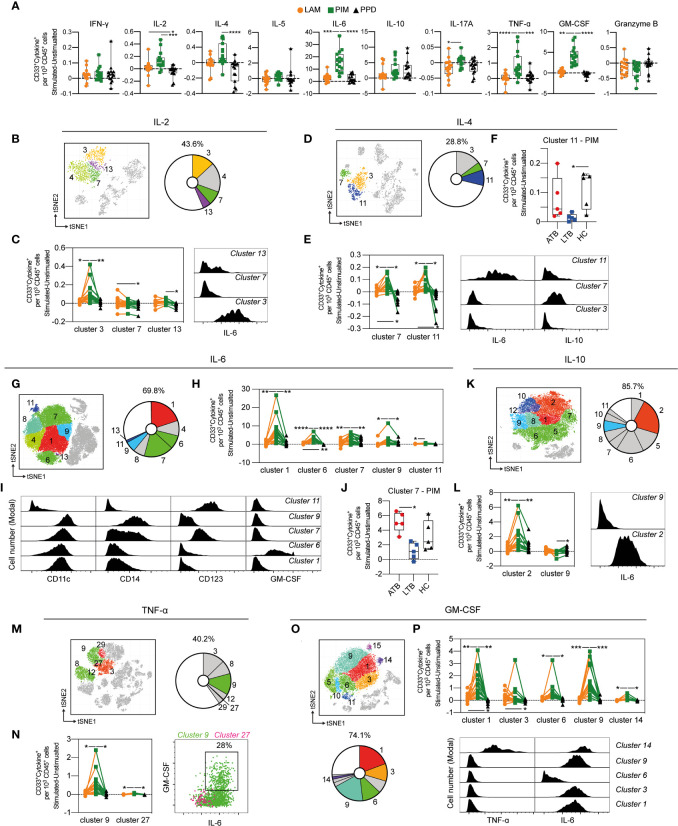
Cytokine production by stimulated CD33^+^ myeloid cells. **(A)** The number of cytokine positive CD33^+^ myeloid cells per 1000 total CD45^+^ cells for each stimulation at 24 h with the background (unstimulated) cytokine-production removed. **(B)** Cluster analysis of IL-2 secreting cells with cluster 3, 4, 7 and 13 corresponding to myeloid cells. **(C)** Clusters significantly affected by stimulation (left) with cluster histograms indicating co-secretion of IL-6. **(D)** Cluster analysis of IL-4 secreting with cluster 3, 7, and 11 corresponding to myeloid cells. **(E)** Clusters significantly affected by stimulation (left) and co-expression with IL-6 and IL-10 (right). **(F)** Comparison of PIM stimulation on donors with active TB (ATB), latent TB (LTB) and healthy controls (HC) in cluster 11. **(G)** Myeloid clusters secreting IL-6 **(H)** significantly affected by stimulation. **(I)** Cell surface phenotype of indicated cluster. **(J)** Differential effect of PIM stimulation on cluster 7 cells in ATB, LTB, and HC. **(K)** Myeloid clusters secreting IL-10 with **(L)** clusters significantly affected by stimulation (left panel) and histograms indicating IL-10 co-expression with IL-6. **(M)** Myeloid clusters secreting TNF-ɑ **(N)** significantly affected by stimulation (left) with co-expression of GM-CSF and IL-6 (right). **(O)** Myeloid clusters secreting GM-CSF. **(P)** Clusters differently affected by stimulation (left) with co-expression of TNF-α and IL-6 (right). Statistical differences between stimulations in **(A)** were evaluated by Friedman’s test with Dunnet’s posttest, while comparisons within multiple clusters **(C, E, H, L, N, P)** were evaluated by a matched-pair two-way ANOVA with Geissner-Greenhouse correction followed by Tukey’s posttest (n=15/stimulation). Groups (ATB/LTB/HC) **(F, J)** were compared using Kruskal-Wallis with Dunn’s posttest (n=5/group) *p<0.05, **p<0.01, ***p<0.001, ****p<0.0001.

The production of cytokines by CD33^+^ myeloid cells was compared for each stimulation (**Figure 8A**). PIM stimulation led to a robust increase of cells producing IL-2, IL-4, IL-6, IL-10, TNF-α, compared to PPD, and of IL-6, IL-17A, TNF-α and GM-CSF in comparison to LAM (**Figure 8A**). Interestingly, IL-10 producing cells were induced by both PIM and PPD (**Figure 8A**), contrasting with the other cytokines that were primarily induced by PIM.

To understand if the effect of stimulation was associated with specific myeloid subsets, we further investigated the impact of stimulation on individual cell clusters. The IL-2 producing myeloid cells constituted 43.6% of all IL2-producing cells and were composed of four different clusters (cluster 3, 4, 7, and 13), of which three were differently affected by the stimuli (**Figure 8B**). For cluster 3 and 13, PIM stimulation led to significantly more IL-2^+^ cells compared with PPD and/or LAM, while cluster 7 was higher in LAM compared to PPD (**Figure 8C**). Cluster 7 expressed CD14, while clusters 3 and 13 were mostly negative for CD14 (**Supplemental Figure 7**). Cluster 3 was associated with the co-production of IL-6 (**Figure 8C**).

Approximately 29% of all IL-4 producing cells after 24 h of stimulation expressed CD33. (**Figure 8D**). These cells were further distributed into three clusters (3, 7, and 11), of which cluster 7 and 11 were significantly higher in number following PIM stimulation compared with LAM and PPD stimulation. LAM stimulation also led to more IL-4 producing cells compared to PPD (**Figure 8E**). Both cluster 7 and 11 produced several other cytokines in addition to IL-4, with cluster 7 also producing IL-10, and cluster 11 producing IL-6, and IL-10 (**Figure 8E**). Interestingly, this effect of multiple cytokine production, was significantly reduced in individuals with LTB compared with ATB and HC (**Figure 8F**).

IL-6 was the most frequent cytokine produced following PIM stimulation (**Figure 8A**). Approximately 70% of all IL-6 secreting cells at 24 h were myeloid cells (**Figure 8G**) with 5 out of 7 clusters showing a significant increase following PIM stimulation compared with PPD and/or LAM (**Figure 8H**). Several subsets of myeloid cells responded with IL-6 production, including CD11c^+^CD14^–^CD123^–^ DCs (cluster 1 and 6), intermediate/non-classical monocytes (CD11c^+^CD14^int/–^CD123^+^, cluster 7), and classical monocytes (CD11c^+^CD14^+^CD123^–^, cluster 9). Among these, the cluster 6 DCs also produced GM-CSF, in addition to IL-6 (**Figure 8I**). Similar to the IL-4^+^IL-6^+^ co-producing cluster 11 (**Figure 8F**), the intermediate/non-classical monocyte cluster 7 contracted in individuals with LTB, compared with those with ATB and HC (**Figure 8J**).

Myeloid cells were the main cell subset identified within IL-10, TNF-α and GM-CSF-producing cells, especially following stimulation with PIM (**Figures 8K, M, O**). One IL-10 cluster, two TNF-α clusters and five GM-CSF clusters were significantly increased compared with LAM and PPD (**Figures 8L, N, P**). Of these, parts of TNF-α cluster 9 and GM-CSF cluster 14 likely corresponded to the same polyfunctional cells as both clusters secreted TNF-α, GM-CSF, and IL-6 (**Figures 8L, N**). From the two IL-10 clusters that were affected by PIM, one only produced IL-10 while the other co-produced IL-6. GM-CSF cluster 1, 3, and 9 also co-produced IL-6, but not TNF-α, while cluster 6 only produced GM-CSF.

In summary, several myeloid cell subsets rapidly responded to stimulation by producing cytokines. The response was primarily induced by PIM and included phenotypes of cells producing both single and multiple cytokines. Among the most polyfunctional responses were cells producing IL-4, IL-6, and IL-10, or TNF-α, GM-CSF and IL-6.

### Stimulation of Myeloid Cells With PIM Is Partially TLR2 Dependent

**Figure 9:**
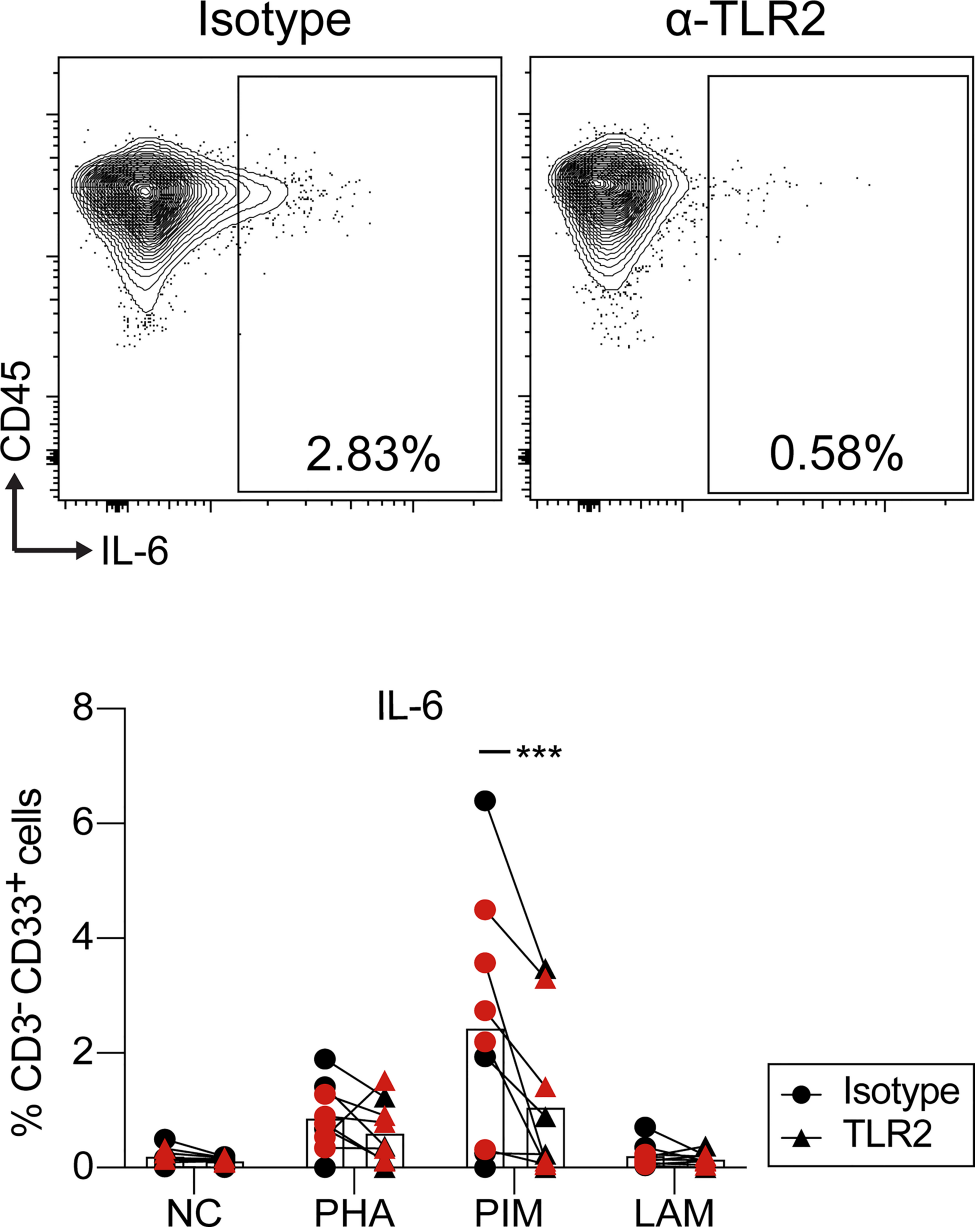
IL-6 production by myeloid cells upon PIM stimulation is regulated *via* TLR2. Dot plots of IL-6 gating within myeloid cells (top) and percentage of cells producing IL-6 within myeloid cells (bottom). PBMCs were pre-treated with anti-TLR2 or isotype control and then stimulated with PIM, LAM, and PHA during 24 h. IL-6 production was evaluated by flow cytometry. Statistical differences between anti-TLR2 and isotype were evaluated by a paired t test (n=9) ***p<0.001. NC, negative control. The red and black colors correspond to two independent experiments.

PIM and LAM stimulation induced a robust immune response in myeloid cells (**Figure 8A**). To investigate the mechanism responsible for this effect, and in particular the dependence on interaction with TLR2, PBMCs from HC were treated with anti-TLR2 blocking antibody before stimulation with PIM, LAM and PHA. Blocking TLR2 led to a reduction in the percentage of CD33^+^IL-6^+^ myeloid cells upon PIM stimulation, but not with LAM or PHA (**Figure 9**). We did not observe any significant effect of blocking TLR2 on IL-6 production from T cells, NK cells, or B cells (data not shown), although the frequency of IL-6^+^ cells was very low on those cell subsets.

## Discussion

In the present study, we show that LAM and PIM induce responses in PBMCs from Mtb-infected individuals that can be distinguished from those obtained from HC. In addition, we show that the responses to these glycolipids are clearly different from those elicited by PPD. The responses involve both expansion and contraction of particular cell subsets and production and secretion of distinct patterns of cytokines and chemokines.

When analyzing intracellular cytokine production, we found that PIM mainly induced antigen-presenting cells to produce a defined set of pro-inflammatory cytokines consisting of IL-2, IL-6, IL-17A, TNF-α and GM-CSF, the anti-inflammatory IL-10 as well as IL-4, but not IFN-γ. LAM triggered responses that tended to be similar to the ones generated by PIM, but weaker in most instances. Classical and intermediate monocytes are known to secrete high levels of pro-inflammatory cytokines in response to microbial products (34). In addition, compared to non-classical monocytes, they were previously shown to exhibit a greater polyfunctional pro-inflammatory response (IL1-α, IL1-β, IL-6, IL-8, IL-10, and TNF-α) to lipomannan from *Mycobacterium smegmatis*, a TLR-2 agonist (34). Here we show that PIM induced multifunctional monocytes producing cytokines in a combination of either pro-inflammatory IL-2, IL-6, GM-CSF and TNF-α, or IL-4 and the anti-inflammatory IL-10. In particular GM-CSF, which is increasingly recognized for its potential role in innate resistance to TB (35), was in our study mainly produced by myeloid cells upon PIM stimulation.

This response contrasted with the quite well-known immune response triggered by PPD, which was dominated primarily by T and NK cells. They produced predominantly the pro-inflammatory cytokines IFN-γ, IL-2, IL-6, IL-17A, TNF-α, and GrzB, but also IL-10, although no IL-4. While T cells simultaneously producing combinations of cytokines have been extensively investigated in the context of the immune response in TB (36–38), we extended these findings to several other cell types. Our results reveal that multiple subsets of myeloid cells, NK, B and T cells respond to glycolipids and/or to PPD, with the production of different combinations of cytokines such as classical functional T cells producing IFN-γ, IL-2 and TNF-α, but also other combinations, such as IL6 and GrzB or IL-17A and TNF-α with or without IFN-γ.

B cells producing IL-10 and GM-CSF are known to be present at relatively low frequencies in human peripheral blood (39, 40). This is in agreement with our finding that B cells were minor producers of IL-10 and GM-CSF, even after stimulation. We did however identify subsets of polyfunctional B cells that produced a combination of GM-CSF, IL-4 and IL-10. These cells were enriched among DN (CD27^–^IgD^–^) B cells and unswitched and switched memory B cells (CD27^+^). The polyfunctional B cells were also approximately 10-fold enriched among B cells expressing CD11c, which was recently associated with B cell activation and formation of atypical B cells (31), also known to expand during ATB (41). Human GM-CSF–expressing B cells are notable for being among the highest producers of both TNF-α and IL-6, and most *in vitro*-induced human IL-10^+^ B cells are also reported to secrete TNF-α and/or IL-6 (42). However, human B cell subsets have been reported to show a near-mutually exclusive expression of GM-CSF and IL-10 (39). By contrast, in our study, B cells stimulated by PIM did not co-produce GM-CSF with TNF-α or IL-6, but rather with IL-4 and IL-10, indicating a different pathway of stimulation.

PIM and LAM did not trigger detectable polyfunctional T cells, although we identified several polyfunctional T cell subsets producing combinations of IFN-γ/IL-2/IL-6/TNF-α/IL-17A that were expanded by PPD stimulation, which is in agreement with previous reports of polyfunctional Mtb-specific T cells producing IFN-γ in combination with IL-2 and TNF-α (36, 37, 43).

One important observation in this study was that upon stimulation with PIM and LAM, cells and supernatants from individuals with ATB or LTB produced less cytokines than the cells from HC. This is obvious for myeloid cells and to a lesser extent for B and T cells. The hyporesponsive state in monocytes in response to PIM and LAM is compatible with trained immunity leading to a tolerogenic cellular response. Trained immunity is defined as a long-term adaptation of innate immune cells leading either to an enhanced responsiveness or a tolerance state to a subsequent challenge (44, 45). Chronic or repeated stimulation through TLRs can render immune cells unresponsive to subsequent challenges with the same or different TLR ligands (46–48) or other bacterial components (49). Our results support the hypothesis that continuous stimulation with LAM and PIM, in ATB and LTB individuals lead to a reduced response to these molecules compared with HC.

The response of myeloid cells to PPD was weaker in LTB compared to HC, indicating hyporesponsiveness also to PPD. This is in line with earlier observations of depression of PPD-induced proliferative responses by monocytes from TB patients (50, 51), where direct stimulation of monocytes primed during Mtb infection appear to be responsible for *in vitro* suppression of PPD responses (50). Interestingly we also found that T cells were somewhat hyporesponsive to PIM. The overall cytokine production was reduced in individuals with ATB upon PIM stimulation. These results are in agreement with the systematic review by Li et al. that found lower levels of IL-17 and IFN-*γ* in ATB when compared to LTB (52). An additional interesting observation in our study was that PIM expanded a cluster of GMCSF^+^ CD8^+^ T cells in HC but not in LTB patients. This hyporesponsiveness to PIM might be caused by T cell exhaustion or tolerance in Mtb infected individuals. Exhaustion of T cells represents a state of functional hyporesponsiveness due to persistent antigen exposure and inflammation reported for TB and other chronic infections (53–55). This effect can also be induced by repeated exposure to mycobacterial antigens (56), including direct exposure of T cells to LAM (57).

Antigen-specific CD4^+^ T-cell activation can be directly inhibited by LAM (58–61) and PIM (59). By interfering with very early events in TCR signaling, LAM and PIM may drive cells to a state of anergy (59, 61), which could provide another explanation of the poor response of cells from ATB and LTB individuals to Mtb glycolipids. Alternatively, the hyporesponsiveness could be indirect, through upstream effects of hyporesponsive myeloid cells, since PIM and LAM also induce proliferation of specific T cells upon presentation by CD1 molecules on myeloid cells.

IL-6 is known to be strongly induced in monocytes and DCs upon TLR2 ligation (62). We observed that PIM stimulation induced IL-6 production mainly in myeloid cells (DCs and classical/nonclassical monocytes). Moreover, treatment with an anti-TLR2 antibody led to partial inhibition of PIM-induced IL-6 production in myeloid cells, suggesting that PIM induces IL-6 production through TLR2. This is in line with other studies where it was observed that PIMs and ManLAM from Mtb induce pro-inflammatory cytokine production in human and mouse Mφs *via* recognition by TLR2 (63–65). However, IL-6 production was not completely abolished suggesting that other mechanisms of PIM stimulation likely remain, or residual IL-6 production may be due to incomplete blocking rather than additional signaling pathways.

LAM and PIM had in general very similar effects, although LAM induced a weaker response than PIM. Presuming that LAM and PIM act through the same TLR2 pathway the different responses are potentially associated with structural differences, where a common active site may be partly masked in LAM compared to PIM. Nigou et al. showed that LAM induces a weaker signal through TLR2 compared to PIM_6_, suggesting that the bulky arabinan domain may mask the mannan chain in such a way that they behave like molecules with a mannan restricted to a single mannosyl unit (65). This is also in line with observations by Shukla et al. that PIM_6_ induces TLR2-mediated extracellular-signal-regulated kinase (ERK) activation and TNF-α secretion in Mφs, while LAM was not an effective functional activator of TLR2 signaling (66). The weaker effect of LAM compared to PIM may also in part depend on the fact that the LAM that was used in the present study has a higher molecular weight compared to PIM resulting in a lower molar concentration.

In contrast to the glycolipids, PPD displayed a markedly different response, mainly by inducing IFN-γ. PPD contains a complex mixture of proteins, including the antigens ESAT-6 and CFP10 that are the antigens used in the Mtb specific IFN-γ release assays. We did not identify which antigens in PPD that were responsible for the immune responses presented in this study. However, since PPD is still widely used in clinical testing, the high level of details presented here may be useful to better understand how individual immune cell subsets react with Mtb proteins.

The hyporesponsive state of monocytes observed in ATB and LTB in response to PIM was more prominent in LTB. The immune profile in LTB is thought to represent a more protective pattern than in ATB (67, 68). It is possible that during LTB a continuous level of stimulation maintains a pool of protective memory cells (18), while at the same time inducing tolerance in monocytes, which could indicate protection of the host from excessive production of pro-inflammatory cytokines and control of lung tissue damage (69).

In conclusion, the detailed high dimensional overview of the cellular source of cytokines produced in response to stimulation with the various antigens, suggesting several novel sources of important cytokines (NK cell and B-cell in particular), will provide a hypothesis- generating resource for future work.

## Data Availability Statement

The raw data supporting the conclusions of this article will be made available by the authors, without undue reservation.

## Ethics Statement

The study was approved by the Regional Ethical Review Board at the Karolinska Institute in Stockholm (approval numbers 2013/1347-31/2 and 2013/2243-31/4) and by the Ethics Committee for Research in Life and Health Sciences of the University of Minho, Portugal (approval number SECVS 014/2015). The patients/participants provided their written informed consent to participate in this study.

## Author Contributions

GK, MC-N, and CS designed the study. CSS, CS, CN, JC-G, and TL performed experiments and/or analysis. EF, GF, and JB included patients and provided clinical data. CSS, CS, and EF generated the figures and tables. BC and PB provided key resources. CSS, CS, EF, JN, MC-N, and GK wrote the first draft and all authors contributed to manuscript revision. All authors contributed to the article and approved the submitted version.

## Funding

The work presented was performed at Karolinska Institutet and Life and Health Sciences Research Institute (ICVS), University of Minho. Financial support was provided by grants from the Swedish Research Council (grant 2016-05683 and 2020-03602) and the Swedish Heart-Lung Foundation (grants 20160336, 20180386, and 20200194) to GK. Grants from Clas Groschinsky’s memorial foundation (M2049), Åke Wiberg’s foundation (M19-0559), the Swedish Medical Association (SLS-934363), and Magnus Bergvall’s foundation (2019-03436) to CS. Grants from the Foundation for Science and Technology (FCT) - project UIDB/50026/2020 and UIDP/50026/2020 to MCN. CSS is supported by an FCT PhD grant, in the context of the Doctoral Program in Applied Health Sciences (PD/BDE/142976/2018). JC-G is supported by an FCT PhD grant, in the context of the Doctoral Program in Aging and Chronic Diseases (PD/BD/137433/2018). CN is a junior researcher under the scope of the FCT Transitional Rule DL57/2016. The funders had no role in study design, data collection and analysis, decision to publish, or preparation of the manuscript.

## Conflict of Interest

The authors declare that the research was conducted in the absence of any commercial or financial relationships that could be construed as a potential conflict of interest.

## Publisher’s Note

All claims expressed in this article are solely those of the authors and do not necessarily represent those of their affiliated organizations, or those of the publisher, the editors and the reviewers. Any product that may be evaluated in this article, or claim that may be made by its manufacturer, is not guaranteed or endorsed by the publisher.

## References

[1] Houben RM Dodd PJ. The Global Burden of Latent Tuberculosis Infection: A Re-Estimation Using Mathematical Modelling. PLoS Med (2016) 13(10):e1002152. doi: 10.1371/journal.pmed.1002152 27780211 PMC5079585

[2] Getahun H Matteelli A Chaisson RE Raviglione M. Latent Mycobacterium Tuberculosis Infection. N Engl J Med (2015) 372(22):2127–35. doi: 10.1056/NEJMra1405427 26017823

[3] Garcia-Vilanova A Chan J Torrelles JB. Underestimated Manipulative Roles of Mycobacterium Tuberculosis Cell Envelope Glycolipids During Infection. Front Immunol (2019) 10:2909. doi: 10.3389/fimmu.2019.02909 31921168 PMC6930167

[4] Kallenius G Correia-Neves M Buteme H Hamasur B Svenson SB. Lipoarabinomannan, and Its Related Glycolipids, Induce Divergent and Opposing Immune Responses to Mycobacterium Tuberculosis Depending on Structural Diversity and Experimental Variations. Tuberc (Edinb) (2016) 96:120–30. doi: 10.1016/j.tube.2015.09.005 26586646

[5] Mazurek J Ignatowicz L Kallenius G Svenson SB Pawlowski A Hamasur B. Divergent Effects of Mycobacterial Cell Wall Glycolipids on Maturation and Function of Human Monocyte-Derived Dendritic Cells. PLoS One (2012) 7(8):e42515. doi: 10.1371/journal.pone.0042515 22880012 PMC3411746

[6] Beatty WL Rhoades ER Ullrich HJ Chatterjee D Heuser JE Russell DG. Trafficking and Release of Mycobacterial Lipids From Infected Macrophages. Traffic (2000) 1(3):235–47. doi: 10.1034/j.1600-0854.2000.010306.x 11208107

[7] Brock M Hanlon D Zhao M Pollock NR. Detection of Mycobacterial Lipoarabinomannan in Serum for Diagnosis of Active Tuberculosis. Diagn Microbiol Infect Dis (2019) 96(2):114937. doi: 10.1016/j.diagmicrobio.2019.114937 31785971

[8] Sakamuri RM Price DN Lee M Cho SN Barry CE 3rd Via LE. Association of Lipoarabinomannan With High Density Lipoprotein in Blood: Implications for Diagnostics. Tuberc (Edinb) (2013) 93(3):301–7. doi: 10.1016/j.tube.2013.02.015 PMC3807251 23507184

[9] Rodriguez ME Loyd CM Ding X Karim AF McDonald DJ Canaday DH. Mycobacterial Phosphatidylinositol Mannoside 6 (PIM6) Up-Regulates TCR-Triggered HIV-1 Replication in CD4+ T Cells. PLoS One (2013) 8(11):e80938. doi: 10.1371/journal.pone.0080938 24282561 PMC3839890

[10] Turner J Torrelles JB. Mannose-Capped Lipoarabinomannan in Mycobacterium Tuberculosis Pathogenesis. Pathog Dis (2018) 76(4). doi: 10.1093/femspd/fty026 PMC5930247 29722821

[11] Vergne I Gilleron M Nigou J. Manipulation of the Endocytic Pathway and Phagocyte Functions by Mycobacterium Tuberculosis Lipoarabinomannan. Front Cell Infect Microbiol (2014) 4:187. doi: 10.3389/fcimb.2014.00187 25629008 PMC4290680

[12] Ernst JD. Macrophage Receptors for Mycobacterium Tuberculosis. Infect Immun (1998) 66(4):1277–81. doi: 10.1128/IAI.66.4.1277-1281.1998 PMC108049 9529042

[13] Liu CH Liu H Ge B. Innate Immunity in Tuberculosis: Host Defense vs Pathogen Evasion. Cell Mol Immunol (2017) 14(12):963–75. doi: 10.1038/cmi.2017.88 PMC5719146 28890547

[14] Drummond RA Brown GD. Signalling C-Type Lectins in Antimicrobial Immunity. PLoS Pathog (2013) 9(7):e1003417. doi: 10.1371/journal.ppat.1003417 23935480 PMC3723563

[15] Macauley MS Crocker PR Paulson JC. Siglec-Mediated Regulation of Immune Cell Function in Disease. Nat Rev Immunol (2014) 14(10):653–66. doi: 10.1038/nri3737 PMC4191907 25234143

[16] Drickamer K Taylor ME. Recent Insights Into Structures and Functions of C-Type Lectins in the Immune System. Curr Opin Struct Biol (2015) 34:26–34. doi: 10.1016/j.sbi.2015.06.003 26163333 PMC4681411

[17] Toyonaga K Torigoe S Motomura Y Kamichi T Hayashi JM Morita YS. C-Type Lectin Receptor DCAR Recognizes Mycobacterial Phosphatidyl-Inositol Mannosides to Promote a Th1 Response During Infection. Immunity (2016) 45(6):1245–57. doi: 10.1016/j.immuni.2016.10.012 27887882

[18] Busch M Herzmann C Kallert S Zimmermann A Hofer C Mayer D. Lipoarabinomannan-Responsive Polycytotoxic T Cells Are Associated With Protection in Human Tuberculosis. Am J Respir Crit Care Med (2016) 194(3):345–55. doi: 10.1164/rccm.201509-1746OC PMC5441105 26882070

[19] Sieling PA Chatterjee D Porcelli SA Prigozy TI Mazzaccaro RJ Soriano T. CD1-Restricted T Cell Recognition of Microbial Lipoglycan Antigens. Science (1995) 269(5221):227–30. doi: 10.1126/science.7542404 7542404

[20] Torrelles JB Sieling PA Zhang N Keen MA McNeil MR Belisle JT. Isolation of a Distinct Mycobacterium Tuberculosis Mannose-Capped Lipoarabinomannan Isoform Responsible for Recognition by CD1b-Restricted T Cells. Glycobiology (2012) 22(8):1118–27. doi: 10.1093/glycob/cws078 PMC3382347 22534567

[21] Fischer K Scotet E Niemeyer M Koebernick H Zerrahn J Maillet S. Mycobacterial Phosphatidylinositol Mannoside Is a Natural Antigen for CD1d-Restricted T Cells. Proc Natl Acad Sci USA (2004) 101(29):10685–90. doi: 10.1073/pnas.0403787101 PMC489995 15243159

[22] Ulrichs T Moody DB Grant E Kaufmann SH Porcelli SA. T-Cell Responses to CD1-Presented Lipid Antigens in Humans With Mycobacterium Tuberculosis Infection. Infect Immun (2003) 71(6):3076–87. doi: 10.1128/IAI.71.6.3076-3087.2003 PMC155760 12761085

[23] Yuan C Qu ZL Tang XL Liu Q Luo W Huang C. Mycobacterium Tuberculosis Mannose-Capped Lipoarabinomannan Induces IL-10-Producing B Cells and Hinders CD4(+)Th1 Immunity. iScience (2019) 11:13–30. doi: 10.1016/j.isci.2018.11.039 30572206 PMC6299163

[24] Chen H Lau MC Wong MT Newell EW Poidinger M Chen J. Cytofkit: A Bioconductor Package for an Integrated Mass Cytometry Data Analysis Pipeline. PLoS Comput Biol (2016) 12(9):e1005112. doi: 10.1371/journal.pcbi.1005112 27662185 PMC5035035

[25] Lundberg M Eriksson A Tran B Assarsson E Fredriksson S. Homogeneous Antibody-Based Proximity Extension Assays Provide Sensitive and Specific Detection of Low-Abundant Proteins in Human Blood. Nucleic Acids Res (2011) 39(15):e102. doi: 10.1093/nar/gkr424 21646338 PMC3159481

[26] Gschwandtner M Derler R Midwood KS. More Than Just Attractive: How CCL2 Influences Myeloid Cell Behavior Beyond Chemotaxis. Front Immunol (2019) 10:2759. doi: 10.3389/fimmu.2019.02759 31921102 PMC6923224

[27] Struyf S Proost P Vandercappellen J Dempe S Noyens B Nelissen S. Synergistic Up-Regulation of MCP-2/CCL8 Activity Is Counteracted by Chemokine Cleavage, Limiting Its Inflammatory and Anti-Tumoral Effects. Eur J Immunol (2009) 39(3):843–57. doi: 10.1002/eji.200838660 19224633

[28] Lin L Finak G Ushey K Seshadri C Hawn TR Frahm N. COMPASS Identifies T-Cell Subsets Correlated With Clinical Outcomes. Nat Biotechnol (2015) 33(6):610–6. doi: 10.1038/nbt.3187 PMC4569006 26006008

[29] Arango Duque G Descoteaux A. Macrophage Cytokines: Involvement in Immunity and Infectious Diseases. Front Immunol (2014) 5:491. doi: 10.3389/fimmu.2014.00491 25339958 PMC4188125

[30] Li B Jones LL Geiger TL. IL-6 Promotes T Cell Proliferation and Expansion Under Inflammatory Conditions in Association With Low-Level RORgammat Expression. J Immunol (2018) 201(10):2934–46. doi: 10.4049/jimmunol.1800016 PMC6324200 30315140

[31] Karnell JL Kumar V Wang J Wang S Voynova E Ettinger R. Role of CD11c(+) T-Bet(+) B Cells in Human Health and Disease. Cell Immunol (2017) 321:40–5. doi: 10.1016/j.cellimm.2017.05.008 28756897

[32] Rubtsov AV Rubtsova K Kappler JW Jacobelli J Friedman RS Marrack P. CD11c-Expressing B Cells Are Located at the T Cell/B Cell Border in Spleen and Are Potent APCs. J Immunol (2015) 195(1):71–9. doi: 10.4049/jimmunol.1500055 PMC4475418 26034175

[33] Reincke ME Payne KJ Harder I Strohmeier V Voll RE Warnatz K. The Antigen Presenting Potential of CD21(low) B Cells. Front Immunol (2020) 11:535784. doi: 10.3389/fimmu.2020.535784 33193306 PMC7609862

[34] de Pablo-Bernal RS Canizares J Rosado I Galva MI Alvarez-Rios AI Carrillo-Vico A. Monocyte Phenotype and Polyfunctionality Are Associated With Elevated Soluble Inflammatory Markers, Cytomegalovirus Infection, and Functional and Cognitive Decline in Elderly Adults. J Gerontol A Biol Sci Med Sci (2016) 71(5):610–8. doi: 10.1093/gerona/glv121 PMC5007736 26286603

[35] Mishra A Singh VK Actor JK Hunter RL Jagannath C Subbian S. GM-CSF Dependent Differential Control of Mycobacterium Tuberculosis Infection in Human and Mouse Macrophages: Is Macrophage Source of GM-CSF Critical to Tuberculosis Immunity? Front Immunol (2020) 11:1599. doi: 10.3389/fimmu.2020.01599 32793233 PMC7390890

[36] Beveridge NE Price DA Casazza JP Pathan AA Sander CR Asher TE. Immunisation With BCG and Recombinant MVA85A Induces Long-Lasting, Polyfunctional Mycobacterium Tuberculosis-Specific CD4+ Memory T Lymphocyte Populations. Eur J Immunol (2007) 37(11):3089–100. doi: 10.1002/eji.200737504 PMC2365909 17948267

[37] Scriba TJ Tameris M Mansoor N Smit E van der Merwe L Isaacs F. Modified Vaccinia Ankara-Expressing Ag85A, a Novel Tuberculosis Vaccine, Is Safe in Adolescents and Children, and Induces Polyfunctional CD4+ T Cells. Eur J Immunol (2010) 40(1):279–90. doi: 10.1002/eji.200939754 PMC3044835 20017188

[38] Wilkinson KA Wilkinson RJ. Polyfunctional T Cells in Human Tuberculosis. Eur J Immunol (2010) 40(8):2139–42. doi: 10.1002/eji.201040731 20853500

[39] Li R Rezk A Miyazaki Y Hilgenberg E Touil H Shen P. Proinflammatory GM-CSF-Producing B Cells in Multiple Sclerosis and B Cell Depletion Therapy. Sci Transl Med (2015) 7(310):310ra166. doi: 10.1126/scitranslmed.aab4176 26491076

[40] Rezk A Li R Bar-Or A. Multiplexed Detection and Isolation of Viable Low-Frequency Cytokine-Secreting Human B Cells Using Cytokine Secretion Assay and Flow Cytometry (CSA-Flow). Sci Rep (2020) 10(1):14823. doi: 10.1038/s41598-020-71750-z 32908164 PMC7481209

[41] Joosten SA van Meijgaarden KE Del Nonno F Baiocchini A Petrone L Vanini V. Patients With Tuberculosis Have a Dysfunctional Circulating B-Cell Compartment, Which Normalizes Following Successful Treatment. PLoS Pathog (2016) 12(6):e1005687. doi: 10.1371/journal.ppat.1005687 27304615 PMC4909319

[42] Lighaam LC Unger PA Vredevoogd DW Verhoeven D Vermeulen E Turksma AW. *In Vitro*-Induced Human IL-10(+) B Cells Do Not Show a Subset-Defining Marker Signature and Plastically Co-Express IL-10 With Pro-Inflammatory Cytokines. Front Immunol (2018) 9:1913. doi: 10.3389/fimmu.2018.01913 30258433 PMC6143818

[43] Darrah PA Patel DT De Luca PM Lindsay RW Davey DF Flynn BJ. Multifunctional TH1 Cells Define a Correlate of Vaccine-Mediated Protection Against Leishmania Major. Nat Med (2007) 13(7):843–50. doi: 10.1038/nm1592 17558415

[44] Netea MG Dominguez-Andres J Barreiro LB Chavakis T Divangahi M Fuchs E. Defining Trained Immunity and Its Role in Health and Disease. Nat Rev Immunol (2020) 20(6):375–88. doi: 10.1038/s41577-020-0285-6 PMC7186935 32132681

[45] Divangahi M Aaby P Khader SA Barreiro LB Bekkering S Chavakis T. Trained Immunity, Tolerance, Priming and Differentiation: Distinct Immunological Processes. Nat Immunol (2021) 22(1):2–6. doi: 10.1038/s41590-020-00845-6 33293712 PMC8020292

[46] Cavaillon JM Adib-Conquy M. Bench-To-Bedside Review: Endotoxin Tolerance as a Model of Leukocyte Reprogramming in Sepsis. Crit Care (2006) 10(5):233. doi: 10.1186/cc5055 17044947 PMC1751079

[47] Nomura F Akashi S Sakao Y Sato S Kawai T Matsumoto M. Cutting Edge: Endotoxin Tolerance in Mouse Peritoneal Macrophages Correlates With Down-Regulation of Surface Toll-Like Receptor 4 Expression. J Immunol (2000) 164(7):3476–9. doi: 10.4049/jimmunol.164.7.3476 10725699

[48] Poovassery JS Vanden Bush TJ Bishop GA. Antigen Receptor Signals Rescue B Cells From TLR Tolerance. J Immunol (2009) 183(5):2974–83. doi: 10.4049/jimmunol.0900495 PMC2789010 19648281

[49] Ifrim DC Quintin J Joosten LA Jacobs C Jansen T Jacobs L. Trained Immunity or Tolerance: Opposing Functional Programs Induced in Human Monocytes After Engagement of Various Pattern Recognition Receptors. Clin Vaccine Immunol (2014) 21(4):534–45. doi: 10.1128/CVI.00688-13 PMC3993125 24521784

[50] Ellner JJ. Suppressor Adherent Cells in Human Tuberculosis. J Immunol (1978) 121(6):2573–9. 309907

[51] Ellner JJ. Regulation of the Human Cellular Immune Response to Mycobacterium Tuberculosis. The Mechanism of Selective Depression of the Response to PPD. Bull Int Union Tuberc Lung Dis (1991) 66(2-3):129–32. 1756294

[52] Li Q Li J Tian J Zhu B Zhang Y Yang K. IL-17 and IFN-Gamma Production in Peripheral Blood Following BCG Vaccination and Mycobacterium Tuberculosis Infection in Human. Eur Rev Med Pharmacol Sci (2012) 16(14):2029–36. 23242733

[53] Schietinger A Greenberg PD. Tolerance and Exhaustion: Defining Mechanisms of T Cell Dysfunction. Trends Immunol (2014) 35(2):51–60. doi: 10.1016/j.it.2013.10.001 24210163 PMC3946600

[54] Blank CU Haining WN Held W Hogan PG Kallies A Lugli E. Defining 'T Cell Exhaustion'. Nat Rev Immunol (2019) 19(11):665–74. doi: 10.1038/s41577-019-0221-9 PMC7286441 31570879

[55] Khan N Vidyarthi A Amir M Mushtaq K Agrewala JN. T-Cell Exhaustion in Tuberculosis: Pitfalls and Prospects. Crit Rev Microbiol (2017) 43(2):133–41. doi: 10.1080/1040841X.2016.1185603 27800700

[56] Liu X Li F Niu H Ma L Chen J Zhang Y. IL-2 Restores T-Cell Dysfunction Induced by Persistent Mycobacterium Tuberculosis Antigen Stimulation. Front Immunol (2019) 10:2350. doi: 10.3389/fimmu.2019.02350 31632413 PMC6783502

[57] Sande OJ Karim AF Li Q Ding X Harding CV Rojas RE. Mannose-Capped Lipoarabinomannan From Mycobacterium Tuberculosis Induces CD4+ T Cell Anergy *via* GRAIL. J Immunol (2016) 196(2):691–702. doi: 10.4049/jimmunol.1500710 26667170 PMC4707121

[58] Karim AF Sande OJ Tomechko SE Ding X Li M Maxwell S. Proteomics and Network Analyses Reveal Inhibition of Akt-mTOR Signaling in CD4(+) T Cells by Mycobacterium Tuberculosis Mannose-Capped Lipoarabinomannan. Proteomics (2017) 17(22). doi: 10.1002/pmic.201700233 PMC5725663 28994205

[59] Mahon RN Rojas RE Fulton SA Franko JL Harding CV Boom WH. Mycobacterium Tuberculosis Cell Wall Glycolipids Directly Inhibit CD4+ T-Cell Activation by Interfering With Proximal T-Cell-Receptor Signaling. Infect Immun (2009) 77(10):4574–83. doi: 10.1128/IAI.00222-09 PMC2747961 19651854

[60] Athman JJ Sande OJ Groft SG Reba SM Nagy N Wearsch PA. Mycobacterium Tuberculosis Membrane Vesicles Inhibit T Cell Activation. J Immunol (2017) 198(5):2028–37. doi: 10.4049/jimmunol.1601199 PMC5322216 28122965

[61] Mahon RN Sande OJ Rojas RE Levine AD Harding CV Boom WH. Mycobacterium Tuberculosis ManLAM Inhibits T-Cell-Receptor Signaling by Interference With ZAP-70, Lck and LAT Phosphorylation. Cell Immunol (2012) 275(1-2):98–105. doi: 10.1016/j.cellimm.2012.02.009 22507872 PMC3352599

[62] Flynn CM Garbers Y Lokau J Wesch D Schulte DM Laudes M. Activation of Toll-Like Receptor 2 (TLR2) Induces Interleukin-6 Trans-Signaling. Sci Rep (2019) 9(1):7306. doi: 10.1038/s41598-019-43617-5 31086276 PMC6513869

[63] Gilleron M Quesniaux VF Puzo G. Acylation State of the Phosphatidylinositol Hexamannosides From Mycobacterium Bovis Bacillus Calmette Guerin and Mycobacterium Tuberculosis H37Rv and Its Implication in Toll-Like Receptor Response. J Biol Chem (2003) 278(32):29880–9. doi: 10.1074/jbc.M303446200 12775723

[64] Riedel DD Kaufmann SH. Differential Tolerance Induction by Lipoarabinomannan and Lipopolysaccharide in Human Macrophages. Microbes Infect (2000) 2(5):463–71. doi: 10.1016/S1286-4579(00)00319-1 10865191

[65] Nigou J Vasselon T Ray A Constant P Gilleron M Besra GS. Mannan Chain Length Controls Lipoglycans Signaling *via* and Binding to TLR2. J Immunol (2008) 180(10):6696–702. doi: 10.4049/jimmunol.180.10.6696 18453589

[66] Shukla S Richardson ET Drage MG Boom WH Harding CV. Mycobacterium Tuberculosis Lipoprotein and Lipoglycan Binding to Toll-Like Receptor 2 Correlates With Agonist Activity and Functional Outcomes. Infect Immun (2018) 86(10):e00450-18. doi: 10.1128/IAI.00450-18 30037791 PMC6204744

[67] Roy Chowdhury R Vallania F Yang Q Lopez Angel CJ Darboe F Penn-Nicholson A. A Multi-Cohort Study of the Immune Factors Associated With M. Tuberculosis Infection Outcomes. Nature (2018) 560(7720):644–8. doi: 10.1038/s41586-018-0439-x PMC6414221 30135583

[68] Lu LL Chung AW Rosebrock TR Ghebremichael M Yu WH Grace PS. A Functional Role for Antibodies in Tuberculosis. Cell (2016) 167(2):433–43.e14. doi: 10.1016/j.cell.2016.08.072 27667685 PMC5526202

[69] Divangahi M Khan N Kaufmann E. Beyond Killing Mycobacterium Tuberculosis: Disease Tolerance. Front Immunol (2018) 9:2976. doi: 10.3389/fimmu.2018.02976 30619333 PMC6305711

