# Supplementary material for: High Dimensional Immune Profiling Reveals Different Response Patterns in Active and Latent Tuberculosis Following Stimulation With Mycobacterial Glycolipids
Source: Front Immunol. 2021 Nov 23;12:727300. doi: 10.3389/fimmu.2021.727300 (PMC8650708; doi:10.3389/fimmu.2021.727300)
Supplement: Supplementary file 1 [file DataSheet_1.docx]

**Supplementary material**

**High dimensional immune profiling reveals different response patterns in active and latent TB following stimulation with mycobacterial glycolipids**

Carolina S. Silva^1,2^*, Christopher Sundling^3,4^*, Elin Folkesson^3,4^, Gabrielle Fröberg^3,4^, Claudia Nobrega^1,2^, João Canto-Gomes^1,2^, Benedict J. Chambers^5^, Tadepally Lakshmikanth^6^, Petter Brodin^6,7^, Judith Bruchfeld^3,4^, Jérôme Nigou^8^, Margarida Correia-Neves^1,2,3^**, Gunilla Källenius^3^**

1. Life and Health Sciences Research Institute, School of Medicine, University of Minho, Braga, Portugal.
2. ICVS/3B's, PT Government Associate Laboratory, Braga, Guimarães, Portugal.
3. Division of Infectious Diseases, Department of Medicine Solna, Center for Molecular Medicine, Karolinska Institutet, Stockholm, Sweden.
4. Department of Infectious Diseases, Karolinska University Hospital, Stockholm, Sweden
5. Center for Infectious Medicine, Department of Medicine, Huddinge, Karolinska Institutet, Stockholm, Sweden.
6. Science for Life Laboratory, Department of Women's and Children's Health, Karolinska Institutet, Stockholm, Sweden.
7. Department of Immunology and Inflammation, Imperial College London, London, UK.
8. Institut de Pharmacologie et de Biologie Structurale, Université de Toulouse, Centre national de la recherche scientifique (CNRS), Université Paul Sabatier, Toulouse, France.

*These two authors contributed equally to this work.

**These two authors contributed equally to this work.

Corresponding author: Christopher Sundling, [christopher.sundling@ki.se](mailto:christopher.sundling@ki.se)

| **Supplemental Table 1. Study individuals** | | | |
| --- | --- | --- | --- |
|  | **ATB (n=5)** | **LTBI (n=5)** | **HC (n=5)** |
| **Male sex** | 5 | 2 | 4 |
| **Age (median, range)** | 39 (24-50) | 39 (25-72) | 25 (22-37) |
| **Comorbidity** | 2* | 0 | 0 |
| **Born in TB high endemic country** | 4 | 3 | 0 |
| **Years since immigration to Sweden** | 8 (0.5-20) | 11 (3-16) | NA |
| **BCG vaccination** | unknown | 3 | 1 |
| **Symptoms** |  |  |  |
| **- cough** | 4 | NA | NA |
| **- fever/night sweats/weight loss** | 3 |  |  |
| **IGRA** |  |  |  |
| **- positive** | NA | 4 | 0 |
| **- conversion (after 2 months)** |  | 1 | 0 |
| **- negative** |  | 0 | 5 |
| **Chest X-Ray/Computed Tomography** |  |  |  |
| **- infiltrates** | 5 | 2** | NA |
| **- cavitaries** | 2 | 0 |  |
| **- pleural effusion** | 3 | 0 |  |
| **Mycobacteriology** |  |  |  |
| **- microscopy positive** | 1 | 0 | NA |
| **- PCR positive** | 1 | 0 |  |
| **- culture positive** | 5 | 0 |  |
| *Well-controlled diabetes mellitus type 2, chronic kidney disease stage 2; **Normal on repeat radiology | | | |
| NA: not applicable; ATB: Active TB; LTB: latent TB; HC: Healthy controls. | | |  |

| **Supplemental Table 2. Broad staining panel for mass cytometry.** | | | | |
| --- | --- | --- | --- | --- |
| **Marker** | **Clone** | **Tag** | **Company** | **Dilution** |
| CD45 | HI30 | 89Y | Fluidigm | 1:200 |
| CD57 | HCD57 | 115In | BioLegend | 1:100 |
| CD19 | HIB19 | 142Nd | BioLegend | 1:100 |
| CD5 | UCHT2 | 143Nd | BioLegend | 1:200 |
| CD16 | 3G8 | 144Nd | BioLegend | 1:100 |
| CD4 | RPA-T4 | 145Nd | BioLegend | 1:100 |
| CD11c | Bu15 | 147Sm | Fluidigm | 1:100 |
| CD123 | 6H6 | 151Eu | BioLegend | 1:100 |
| CD3e | UCHT1 | 154Sm | Fluidigm | 1:200 |
| CD14 | M5E2 | 160Gd | BioLegend | 1:100 |
| CD161 | HP-3G10 | 161Dy | BioLegend | 1:100 |
| CD127 | A019D5 | 165Ho | Fluidigm | 1:100 |
| CD38 | HIT2 | 168Er | BioLegend | 1:100 |
| CD45RA | HI100 | 169Tm | Fluidigm | 1:100 |
| CD20 | 2H7 | 170Er | BioLegend | 1:100 |
| IgD | IA6-2 | 172Yb | BioLegend | 1:100 |
| Cell-ID™ Intercalator-Ir | DNAIr-191 | - | Fluidigm | 1:1000 |
| Cell-ID™ Intercalator-Ir | DNAIr-193 | - | Fluidigm | 1:1000 |
| CD31 | WM59 | 148Nd | BioLegend | 1:200 |
| HLA-DR | L243 | 163Dy | BioLegend | 1:200 |
| CD44 | BJ18 | 174Yb | BioLegend | 1:100 |
| CD8a | SK1 | 146Nd | BioLegend | 1:300 |
| CD11b (MAC1) | Mac-1 | 209Bi | Fluidigm | 1:200 |
| CD56 | NCAM16.2 | 173Yb | BD | 1:200 |
| CD7 | CD7-687 | 155Gd | BioLegend | 1:200 |
| CD27 | L128 | 167Er | Fluidigm | 1:200 |
| Siglec-8 | 7C9 | 164Dy | Fluidigm | 1:100 |
| CD33 | WM53 | 157Gd | BioLegend | 1:100 |
| TCRγδ | 5A6.E9 | 152Sm | Fisher | 1:100 |

| **Supplemental Table 3. Intracellular staining panel for mass cytometry.** | | | | | |
| --- | --- | --- | --- | --- | --- |
| **Marker** | **Clone** | **Tag** | **Company** | **Dilution** |  |
| IL-2 | MQ1-17H12 | 158Gd | Fluidigm | 1:200 |  |
| IL-4 | MP4-25D2 | 149Sm | BioLegend | 1:100 |  |
| IL-5 | TRFK5 | 153Eu | BioLegend | 1:100 |  |
| IL-6 | MQ2-13AS | 156Gd | Fluidigm | 1:100 |  |
| IL-10 | JES3-9D7 | 159Tb | BioLegend | 1:100 |  |
| IL-17A | N49-653 | 166Er | BioLegend | 1:100 |  |
| IFN-γ | B27 | 150Nd | BioLegend | 1:125 |  |
| TNF | MAb11 | 175Lu | BioLegend | 1:75 |  |
| Granzyme B | GB11 | 171Yb | Fluidigm | 1:100 |  |
| GM-CSF | BVD2-21C11 | 176Yb | BioLegend | 1:75 |  |

| **Supplemental Table 4. Olink inflammation panel.** | | | |
| --- | --- | --- | --- |
| **Protein** | **UniProt ID** | **Protein** | **UniProt ID** |
| Adenosine Deaminase (ADA) | P00813 | Fractalkine (CX3CL1) | P78423 |
| Artemin (ARTN) | Q5T4W7 | Glial cell line-derived neurotrophic factor (GDNF) | P39905 |
| Axin-1 (AXIN1) | O15169 | Hepatocyte growth factor (HGF) | P14210 |
| Beta-nerve growth factor (Beta-NGF) | P01138 | Interferon gamma (IFN-gamma) | P01579 |
| Caspase-8 (CASP-8) | Q14790 | Interleukin-1 alpha (IL-1 alpha) | P01583 |
| C-C motif chemokine 3 (CCL3) | P10147 | Interleukin-2 (IL-2) | P60568 |
| C-C motif chemokine 4 (CCL4) | P13236 | Interleukin-2 receptor subunit beta (IL-2RB) | P14784 |
| C-C motif chemokine 19 (CCL19) | Q99731 | Interleukin-4 (IL-4) | P05112 |
| C-C motif chemokine 20 (CCL20) | P78556 | Interleukin-5 (IL5) | P05113 |
| C-C motif chemokine 23 (CCL23) | P55773 | Interleukin-6 (IL6) | P05231 |
| C-C motif chemokine 25 (CCL25) | O15444 | Interleukin-7 (IL-7) | P13232 |
| C-C motif chemokine 28 (CCL28) | Q9NRJ3 | Interleukin-8 (IL-8) | P10145 |
| CD40L receptor (CD40) | P25942 | Interleukin-10 (IL10) | P22301 |
| CUB domain-containing protein 1 (CDCP1) | Q9H5V8 | Interleukin-10 receptor subunit alpha (IL-10RA) | Q13651 |
| C-X-C motif chemokine 1 (CXCL1) | P09341 | Interleukin-10 receptor subunit beta (IL-10RB) | Q08334 |
| C-X-C motif chemokine 5 (CXCL5) | P42830 | Interleukin-12 subunit beta (IL-12B) | P29460 |
| C-X-C motif chemokine 6 (CXCL6) | P80162 | Interleukin-13 (IL-13) | P35225 |
| C-X-C motif chemokine 9 (CXCL9) | Q07325 | Interleukin-15 receptor subunit alpha (IL-15RA) | Q13261 |
| C-X-C motif chemokine 10 (CXCL10) | P02778 | Interleukin-17A (IL-17A) | Q16552 |
| C-X-C motif chemokine 11 (CXCL11) | O14625 | Interleukin-17C (IL-17C) | Q9P0M4 |
| Cystatin D (CST5) | P28325 | Interleukin-18 (IL-18) | Q14116 |
| Delta and Notch-like epidermal growth factor-related receptor (DNER) | Q8NFT8 | Interleukin-18 receptor 1 (IL-18R1) | Q13478 |
| Eotaxin (CCL11) | P51671 | Interleukin-20 (IL-20) | Q9NYY1 |
| Eukaryotic translation initiation factor 4E-binding protein 1 (4E-BP1) | Q13541 | Interleukin-20 receptor subunit alpha (IL-20RA) | Q9UHF4 |
| Fibroblast growth factor 21 (FGF-21) | Q9NSA1 | Interleukin-22 receptor subunit alpha-1 (IL-22 RA1) | Q8N6P7 |
| Fibroblast growth factor 23 (FGF-23) | Q9GZV9 | Interleukin-24 (IL-24) | Q13007 |
| Fibroblast growth factor 5 (FGF-5) | P12034 | Interleukin-33 (IL-33) | O95760 |
| Fibroblast growth factor 19 (FGF-19) | O95750 | Latency-associated peptide transforming growth factor beta-1 (LAP TGF-beta-1) | P01137 |
| Fms-related tyrosine kinase 3 ligand (Flt3L) | P49771 | Leukemia inhibitory factor (LIF) | P15018 |
| Leukemia inhibitory factor receptor (LIF-R) | P42702 | STAM-binding protein (STAMBP) | O95630 |
| Macrophage colony-stimulating factor 1 (CSF-1) | P09603 | Stem cell factor (SCF) | P21583 |
| Matrix metalloproteinase-1 (MMP-1) | P03956 | Sulfotransferase 1A1 (ST1A1) | P50225 |
| Matrix metalloproteinase-10 (MMP-10) | P09238 | T cell surface glycoprotein CD6 isoform (CD6) | P30203 |
| Monocyte chemotactic protein 1 (MCP-1) | P13500 | T-cell surface glycoprotein CD5 (CD5) | P06127 |
| Monocyte chemotactic protein 2 (MCP-2) | P80075 | T-cell surface glycoprotein CD8 alpha chain (CD8A) | P01732 |
| Monocyte chemotactic protein 3 (MCP-3) | P80098 | Thymic stromal lymphopoietin (TSLP) | Q969D9 |
| Monocyte chemotactic protein 4 (MCP-4) | Q99616 | TNF-beta (TNFB) | P01374 |
| Natural killer cell receptor 2B4 (CD244) | Q9BZW8 | TNF-related activation-induced cytokine (TRANCE) | O14788 |
| Neurotrophin-3 (NT-3) | P20783 | TNF-related apoptosis-inducing ligand (TRAIL) | P50591 |
| Neurturin (NRTN) | Q99748 | Transforming growth factor alpha (TGF-alpha) | P01135 |
| Oncostatin-M (OSM) | P13725 | Tumor necrosis factor (Ligand) superfamily, member 12 (TWEAK) | O43508 |
| Osteoprotegerin (OPG) | O00300 | Tumor necrosis factor (TNF) | P01375 |
| Programmed cell death 1 ligand 1 (PD-L1) | Q9NZQ7 | Tumor necrosis factor ligand superfamily member 14 (TNFSF14) | O43557 |
| Protein S100-A12 (EN-RAGE) | P80511 | Tumor necrosis factor receptor superfamily member 9 (TNFRSF9) | Q07011 |
| Signaling lymphocytic activation molecule (SLAMF1) | Q13291 | Urokinase-type plasminogen activator (uPA) | P00749 |
| SIR2-like protein 2 (SIRT2) | Q8IXJ6 | Vascular endothelial growth factor A (VEGF-A) | P15692 |

| **Supplemental Table 5. Membrane and intracellular staining panel for flow cytometry.** | | | | | |
| --- | --- | --- | --- | --- | --- |
| **Marker** | **Clone** | **Fluorochrome** | **Company** | **Dilution** |  |
| **Surface staining** | | | | |  |
| CD3 | UCHT1 | V500 | BD Horizon | 1:10 |  |
| CD4 | HI30 | APC-Cy7 | BioLegend | 1:80 |  |
| CD8 | SK1 | BV650 | BioLegend | 1:40 |  |
| CD19 | HIB19 | BV711 | BioLegend | 1:40 |  |
| CD33 | WM53 | PE-Cy7 | BioLegend | 1:40 |  |
| CD45 | HI30 | BV605 | BioLegend | 1:10 |  |
| CD56 | NCAM16.2 | BV786 | BD Horizon | 1:10 |  |
| **Viability staining** | | | | |  |
| Fixable viability dye | - | eFluor™ 450 | eBiosciences | 1:1000 |  |
| **Intracellular staining** | | | | |  |
| IL-2 | MQ1-17H12 | PerCP-Cy5.5 | BioLegend | 1:20 |  |
| IL-6 | MQ2-13AS | FITC | Fluidigm | 1:7 |  |
| TNF | MAb11 | PE | BioLegend | 1:20 |  |
| GM-CSF | BVD2-21C11 | APC | BioLegend | 1:10 |  |


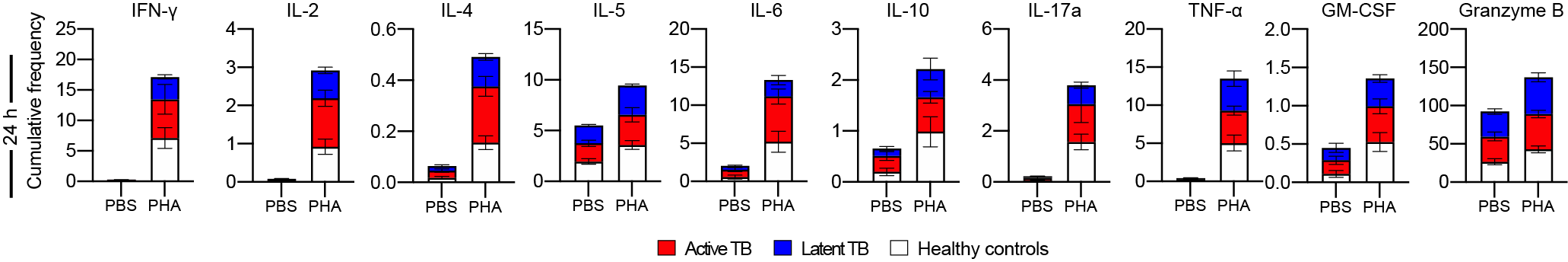


**Supplemental Figure 1. Intracellular cytokine staining after PHA stimulation.**

Frequency of cytokine-producing leukocytes upon PHA stimulation for 24 h. All donors merged (n=15/condition).


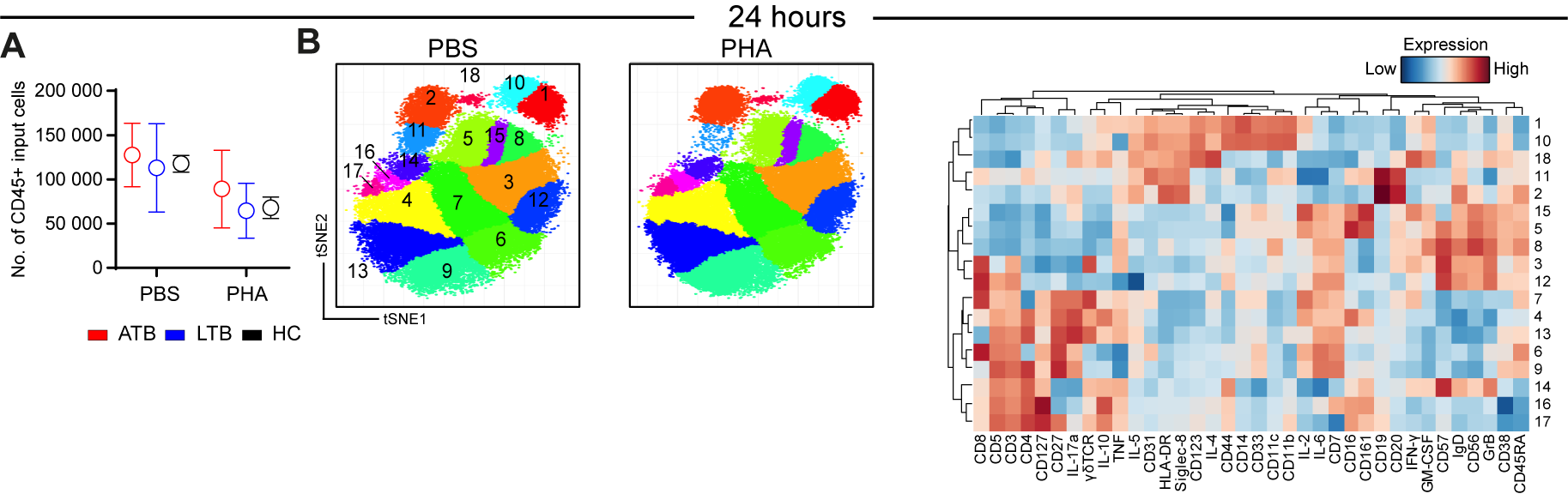


**Supplemental Figure 2. Clustering analysis of total leukocytes after 24 h of stimulation.**

Numbers of CD45^+^ input cells in unstimulated (PBS) and PHA-stimulated PBMCS in ATB, LTB, and HC, after 24 h of stimulation. **(B)** Cluster identification and median heatmap expression of each marker.


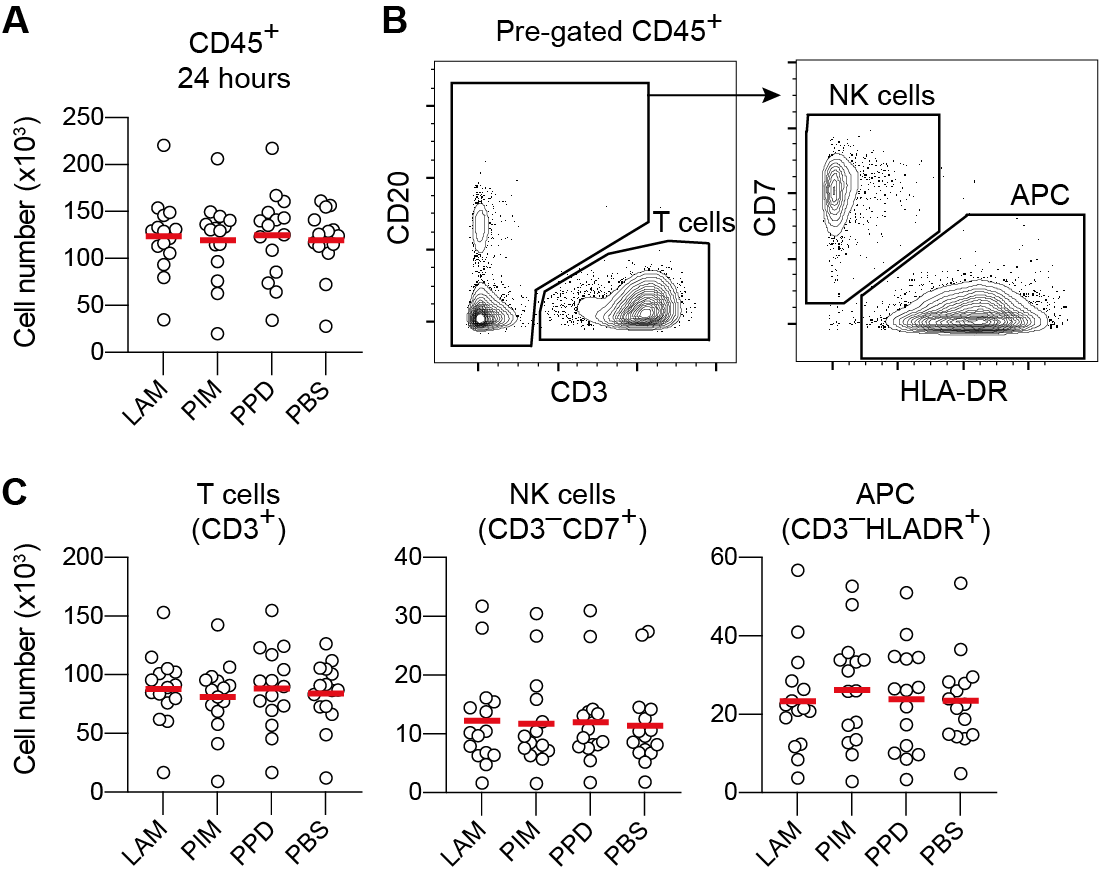


**Supplemental Figure 3. Effect of stimulation with LAM, PIM, and PPD on culture cell numbers.**

(**A**) Number of total CD45^+^ cells for each condition at 24 h of stimulation. (**B**) Representative gating strategy to further subdivide CD45^+^ cells into CD3^+^ T cells, CD3^–^CD7^+^ NK cells, and CD3^–^CD7^–^HLADR^+^ antigen-presenting cells (APC). (**C**) Number of T cells, NK cells and APCs after 24 h. Statistics was evaluated by Friedmans test with Dunn’s posttest where every group was compared with unstimulated cells (PBS).


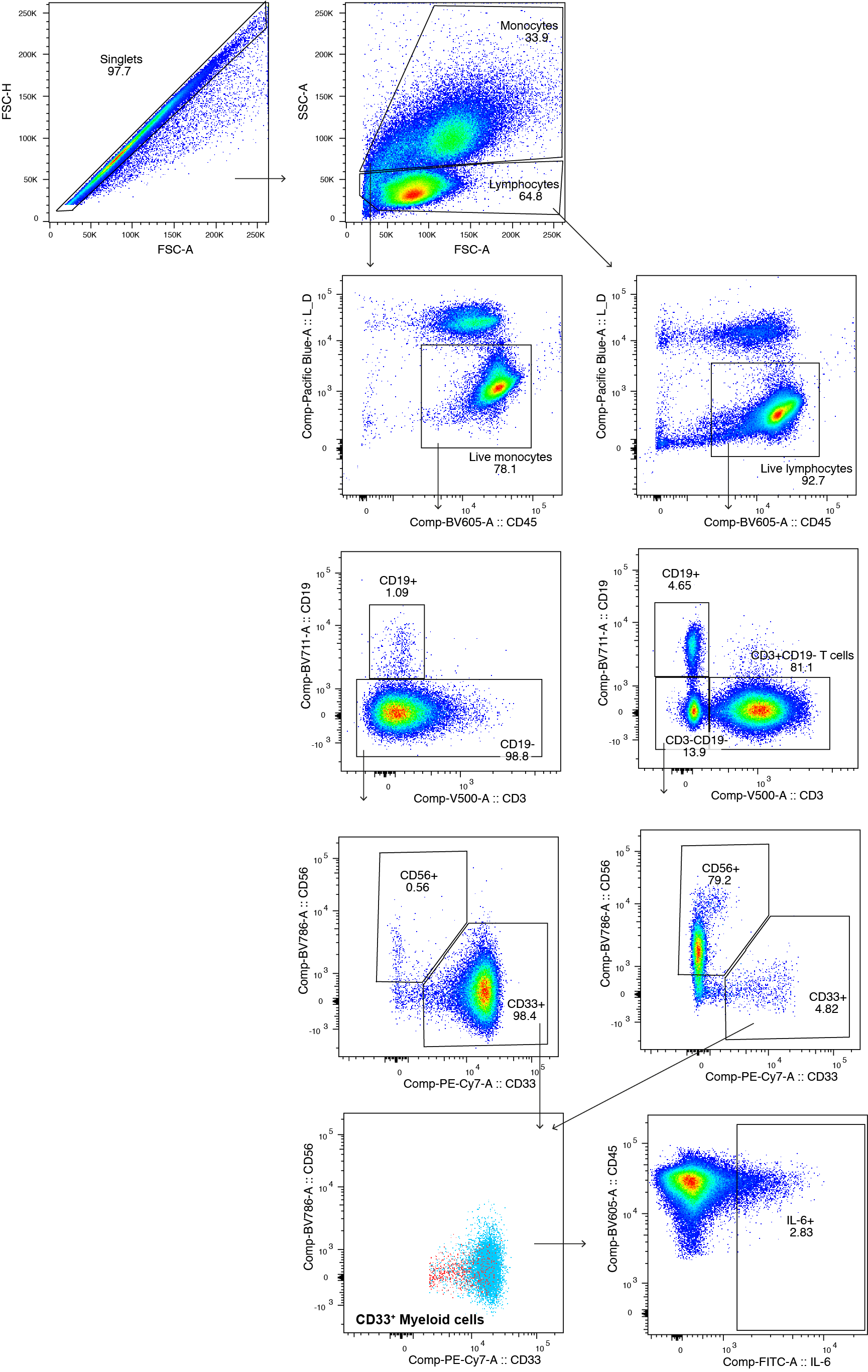


**Supplemental figure 4. Flow cytometry gating strategy for the identification of IL-6-producing-myeloid cells.** Total lymphocytes and monocytes were gated from singlets (based on FSC-H/FSC-A) based on SSC-S/FSC-A. Live lymphocytes and live monocytes were gated from total lymphocytes and total monocytes, respectively. Myeloid cells (CD3^–^CD33^+^) were gated on CD3^–^CD19^–^ both in lymphocytes and monocytes gate and merged using a Boolean gate. IL-6 producing cells were gated on myeloid cells.


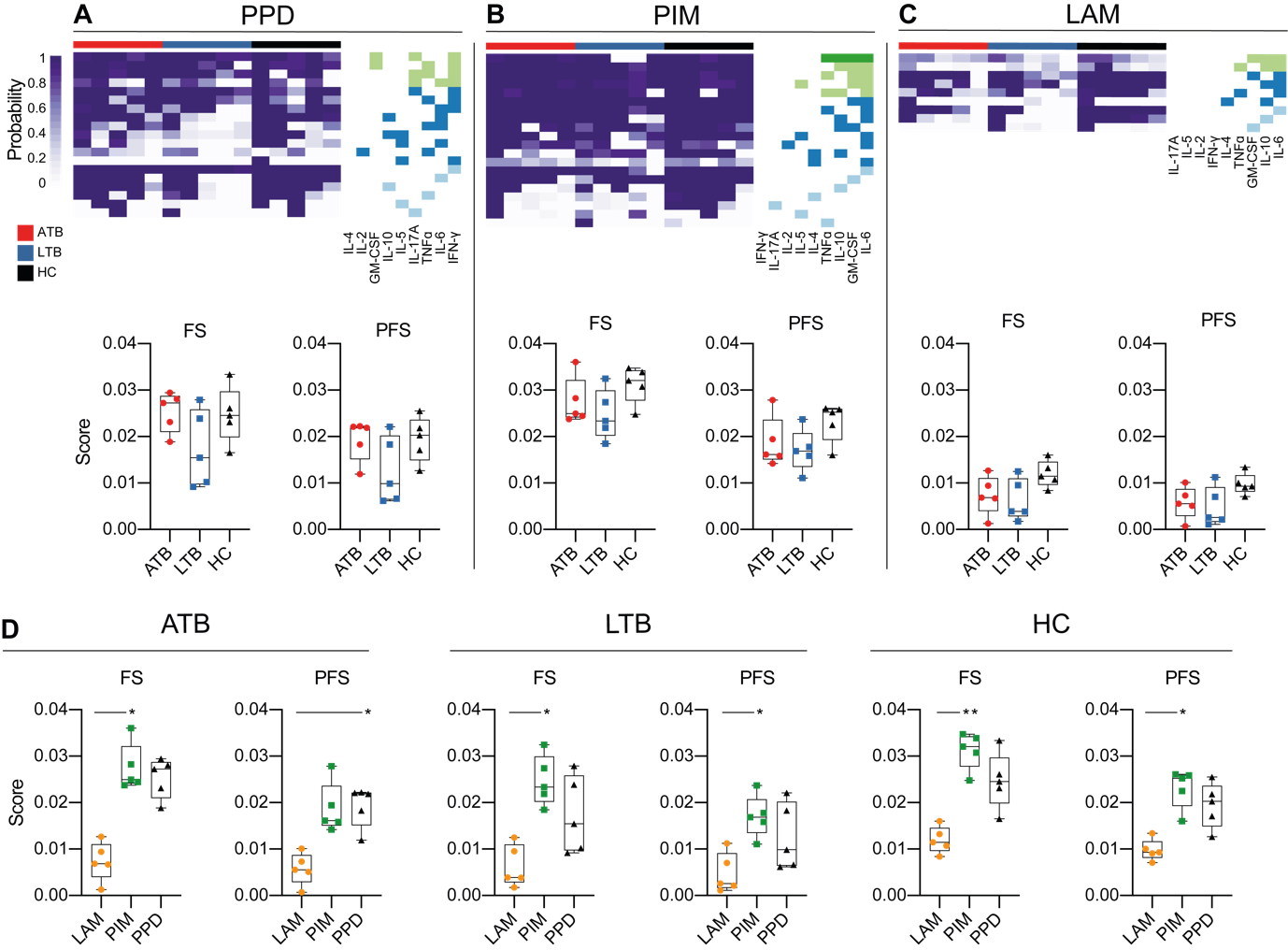


**Supplemental figure 5. Polyfunctionality analysis of CD45^+^ cells in response to PPD, PIM, and LAM stimulations.** Boolean gates were created from the nine individual cytokines in FlowJo to divide responding cells into 512 distinct subsets corresponding to all possible combinations of cytokines. These data were then analyzed using COMPASS in R studio. Only subsets having over five cells in more than two subjects were included in the analysis. Heatmaps of COMPASS posterior probability for stimulation with (**A**) PPD (top), (**B**) PIM (top), and (**C**) LAM (top). Rows correspond to the different cell subsets modeled by COMPASS, where each color represent different levels of functionality, with one cytokine (light blue), two cytokines (dark blue), three cytokines (light green), and four cytokines (dark green). Columns correspond to individual donors. Each cell of the heatmap shows the probability that a given cell-subset (row) has a stimulation-specific response in the corresponding subject (column), where the probability is color-coded from zero (white) to one (purple). Box plots of Functionality score (FS) and polyfunctionality score (PFS) (**A**, **B**, and **C** – bottom panels) stratified by groups and (**D**) stimulations. The FS is defined as the proportion of stimulation-specific subsets detected among all possible combinations, while PFS weighs the different subsets by their degree of functionality. Statistical evaluation between groups in (**A, B, and C**) were done by Kruskal-Wallis, while comparisons between stimulations in (**D**) were evaluated by Friedman’s test with Dunns’ posttest *p<0.05, **p<0.01.


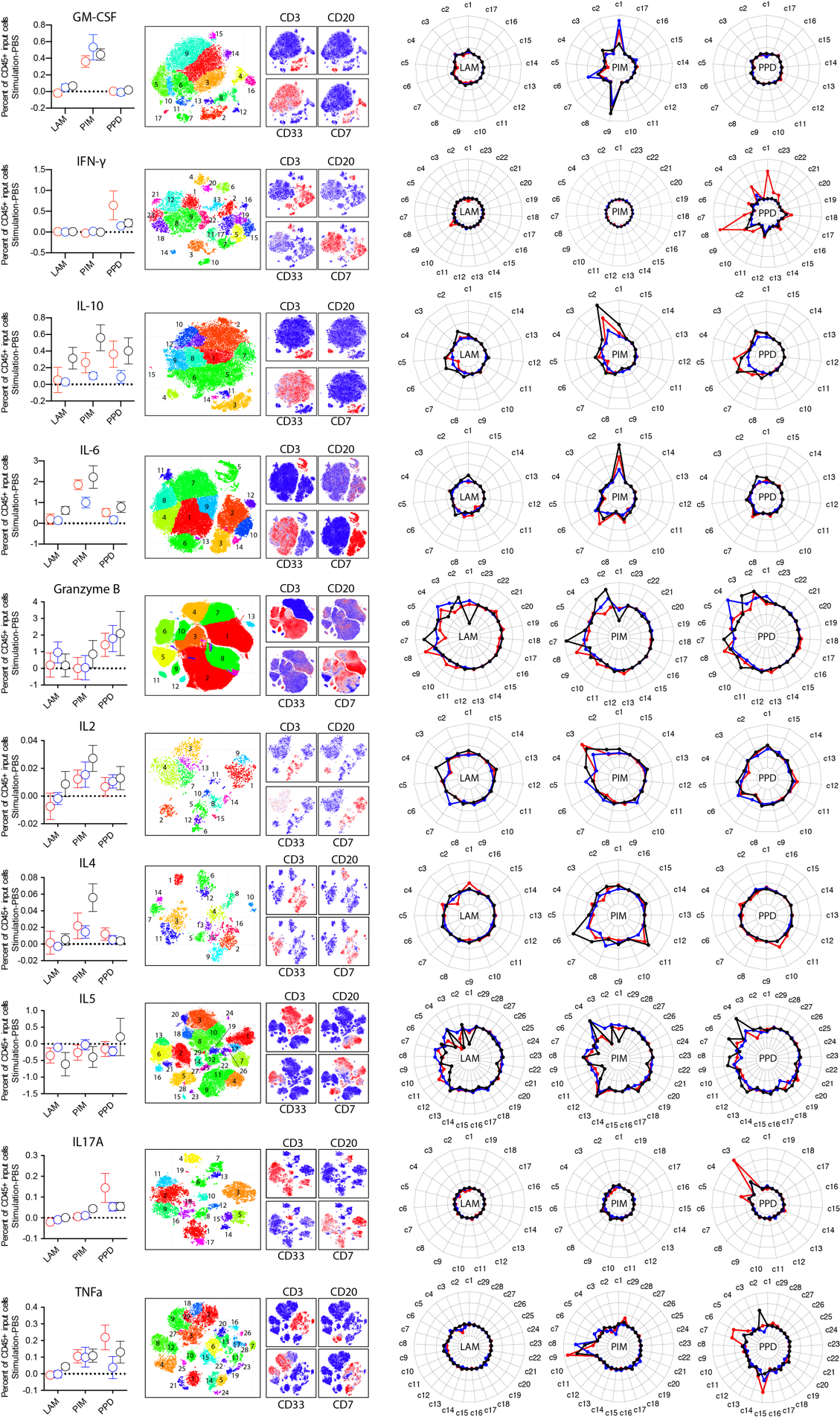


**Supplemental Figure 6. Group and cluster analysis of cytokine^+^ cells after 24 h of stimulation.**

Cytokine^+^ cells were exported after 24 h of stimulation as gated in figure 3 and analyzed by tSNE dimensionality reduction and clustering using cytofkit with ClusterX. To provide some guidance on the type of cells that compose the different clusters the expression of CD3 (T cells), CD20 (B cells), CD33 (myeloid cells), and CD7 (NK cells if negative for CD3) is shown in the tSNE plot. Red indicates high expression and blue indicates low expression. The frequency of cytokine^+^ cells for each stimulation minus background signal (PBS) grouped (left graphs, mean +/– SEM) and from each cluster (spider plots, mean) out of total CD45+ cells, were compared between individuals with active TB (red), latent TB (blue), and healthy controls (black).


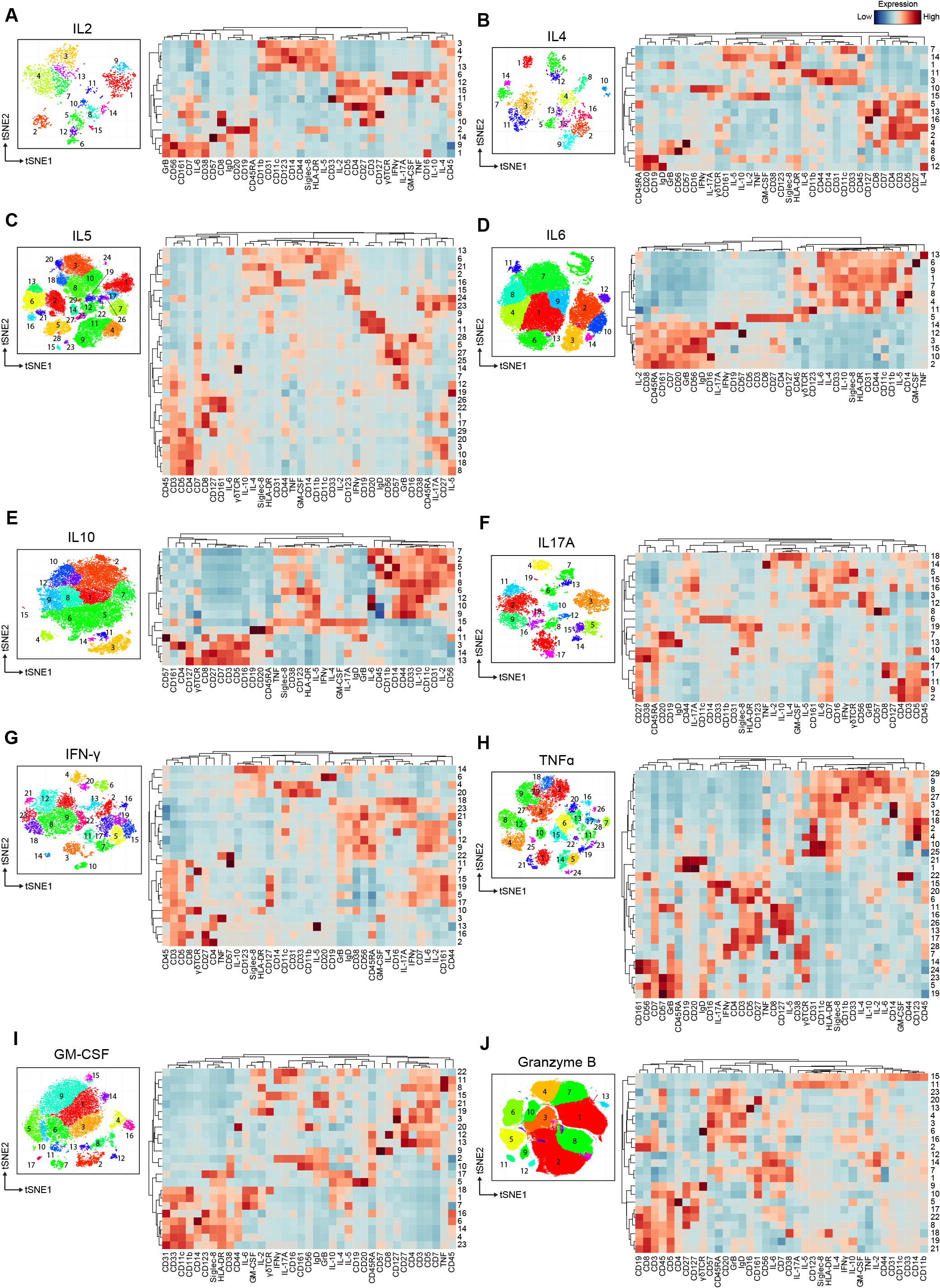


**Supplemental Figure 7. Marker expression of cytokine^+^ cells after 24 h of stimulation.**

Cluster identification with heatmap indicating median expression level for each marker (Y-axis indicates each cluster; X-axis indicates each marker) in **(A)** IL-2, **(B)** IL-4, **(C)** IL-5, **(D)** IL-6, **(E)** IL-10, **(F)** IL-17A, **(G)** IFN-γ, **(H)** TNF-ɑ, **(I)** GM-CSF, and **(J)** granzyme B-producing cells. The expression is normalized within the observed range for each marker.
